# Usage Frequency and Ecotoxicity of Skin Depigmenting Agents

**DOI:** 10.3390/ph18030368

**Published:** 2025-03-04

**Authors:** Sandra Mota, Liliana Rego, Emília Sousa, Maria Teresa Cruz, Isabel Martins de Almeida

**Affiliations:** 1 Associate Laboratory i4HB—Institute for Health and Bioeconomy, Faculty of Pharmacy, University of Porto, R. Jorge Viterbo Ferreira 228, 4050-313 Porto, Portugal; up201608486@up.pt (S.M.); up201203377@edu.ff.up.pt (L.R.); 2 UCIBIO—Applied Molecular Biosciences Unit, Department of Drug Sciences, Faculty of Pharmacy, University of Porto, R. Jorge Viterbo Ferreira 228, 4050-313 Porto, Portugal; 3 Departamento de Ciências Químicas, Faculdade de Farmácia, Universidade do Porto, R. Jorge Viterbo Ferreira 228, 4050-313 Porto, Portugal; esousa@ff.up.pt; 4 CIIMAR—Centro Interdisciplinar de Investigação Marinha e Ambiental, Avenida General Norton de Matos, S/N, 4450-208 Matosinhos, Portugal; 5 Faculty of Pharmacy, University of Coimbra, Azinhaga de Santa Comba, 3000-548 Coimbra, Portugal; trosete@ff.uc.pt; 6 CIBB—Center for Innovative Biomedicine and Biotechnology, CNC—Center for Neuroscience and Cell Biology, University of Coimbra, 3004-504 Coimbra, Portugal

**Keywords:** depigmenting agents, whitening, cosmetics, usage frequency, ecotoxicity, aquatic toxicity

## Abstract

**Background/Objectives:** Depigmenting cosmetic products are a fast-growing segment of the health products market, driven by consumer demand to address skin hyperpigmentation. Simultaneously, interest in products with a reduced environmental impact is increasing. However, the potential environmental risks, especially in aquatic ecosystems, of depigmenting products remain unexplored. This study assesses the usage frequency of skin depigmenting agents in cosmetic products and compiles data on the biodegradability and acute aquatic toxicity of the most prevalent compounds. **Methods:** A market analysis of Portuguese pharmacies and parapharmacies in 2022 identified prevalent depigmenting agents. Scientific evidence on their biodegradability and acute aquatic toxicity was compiled, and when data was unavailable, in silico predictions were conducted. **Results:** The study identified the ten most-used depigmenting agents in cosmetic products, including hydroxy/keto acids, as well as vitamin C and derivatives, with a usage frequency surpassing 50%. While most were naturally derived and showed low environmental risk, synthetic and highly lipophilic depigmenting agents found in 35 of 70 products (ascorbyl tetraisopalmitate/tetrahexyldecyl ascorbate and resorcinol derivatives) showed a higher potential for environmental hazard. **Conclusions:** The findings underscore the need for further research on the presence of these cosmetic ingredients in aquatic ecosystems and a reassessment of regulatory frameworks concerning their environmental impact. Mitigation strategies should emphasize biodegradable alternatives, renewable sources, and molecular modifications to reduce toxicity while maintaining depigmenting efficacy and skin safety. This study provides original insights into commonly used depigmenting agents in the health products market and their chemical structures, offering valuable opportunities for innovation in chemical/pharmaceutical industries.

## 1. Introduction

Skin depigmenting agents, found in cosmetic products, chemical peels, and topical or oral drugs, are commonly used to treat hyperpigmentation [1,2]. Hyperpigmentation can result from altered melanin production, which can lead to skin pigmentation problems, namely melasma, senile/solar lentigo, age spots, post-inflammatory hyperpigmentation, or pigmented scars [3]. The main causes for these abnormalities are exposure to UV radiation, chemicals or drugs like antibiotics and chemotherapy drugs, and disease-related hyperpigmentation [4,5].

Melanin, the pigment responsible for the pigmentation of the skin, hair, and eyes, also plays an important role in ultraviolet (UV) protection [1,4,6]. It is produced by cells located in the inner layer of the epidermis—melanocytes—through a process called melanogenesis [6]. When the skin is exposed to UV radiation, particularly UVB, tyrosine is oxidized to dopaquinone through the enzyme tyrosinase, which starts the process of melanogenesis [7]. Tyrosinase is a copper-containing oxidase that is only found in melanocytes [4]. At the end of the chemical and enzymatic processes, two types of melanin can be obtained depending on the presence or absence of cysteine, respectively, pheomelanin (red/yellow) and eumelanin (brown/black) [6,7].

Skin depigmenting agents target several stages of this melanogenesis process. They can function through various mechanisms, including tyrosinase inhibition, suppression of tyrosinase gene transcription, acceleration of epidermal cell turnover, and by exhibiting antioxidant or anti-inflammatory properties [2,6]. By targeting the production of melanin, depigmenting agents help to lighten skin and treat hyperpigmentation issues.

**Figure 1:**
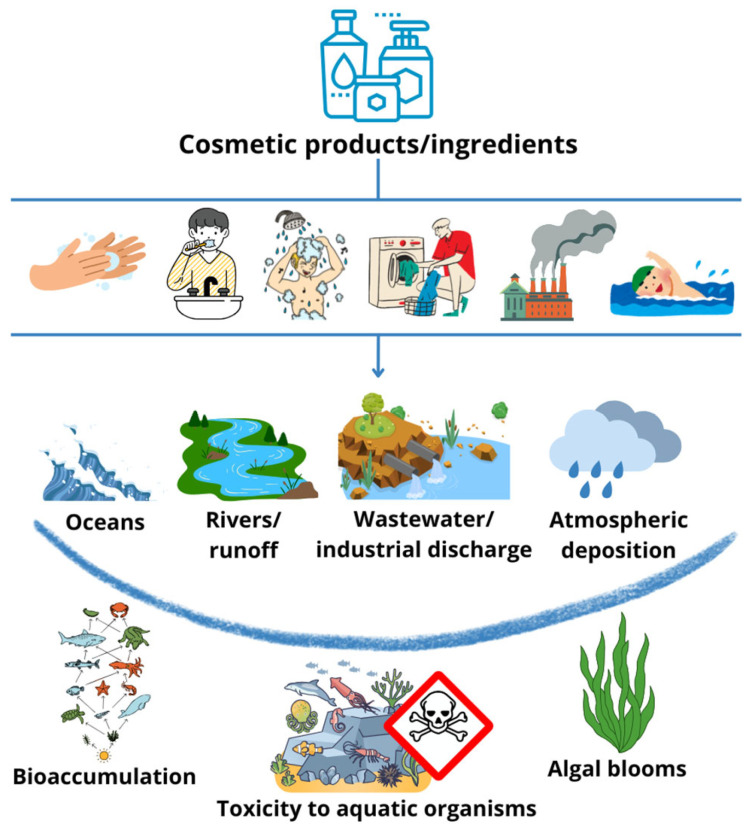
Routes of cosmetic ingredient contamination in aquatic systems and the resulting ecological effects.

The demand for skin-lightening cosmetic products has grown due to the efficacy of these compounds in treating hyperpigmentation issues and in response to the rising prevalence of pigmentary disorders, particularly among individuals with darker skin tones, as well as the increasing consumer preferences for even skin tones [1,8,9]. In particular, Asia is the region with the largest market share for this type of product, with an estimated 15% of the global population investing in them [1]. Additionally, consumers are progressively seeking more sustainable and lower environmental impact products [10]. As the market for skin depigmenting products continues to expand, evaluating their environmental impact is important, particularly due to their release into aquatic ecosystems through several ways and their potential toxic effects (Figure 1).

The environmental footprint of the cosmetics industry is considerable and must be taken into account at each stage of the cosmetic product life cycle [11]. For instance, the highest environmental impact of cosmetic products is in the consumer use and post-use phases, which are directly related to the contamination of aquatic environments [11]. Therefore, cosmetic formulations must contain ingredients with reduced toxicity to aquatic systems. Currently, there are a lack of studies on the market prevalence of skin depigmenting agents combined with their ecotoxicological profiles, and thus this subject requires further investigation. Considering this, the aim of this study is to provide an original market analysis by characterizing the frequency of use of skin depigmenting agents in 70 commercial cosmetic products and providing the current scientific knowledge regarding their acute aquatic toxicity. Biodegradability data of the most commonly used depigmenting agents were also collected, and, if not available, an in silico biodegradability prediction was carried out to support the evidence about their aquatic toxicity. The findings of this study are relevant to regulatory frameworks, the sustainability of the cosmetics industry, and environmental safety. Furthermore, this study aligns with global environmental policies, including the EU Chemicals Strategy for Sustainability and the Green Deal, which emphasize the need for safer and more sustainable use of chemical substances.

## 2. Results and Discussion

### 2.1. Overview of the Use of Depigmenting Agents in Cosmetic Products

**Figure 2:**
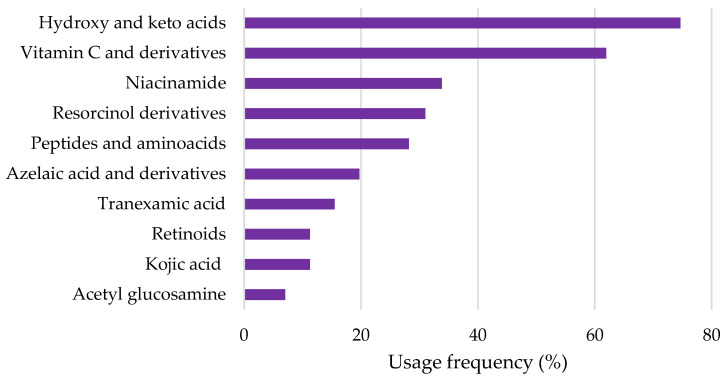
Top 10 depigmenting agents in cosmetic products.

A total of 58 pure depigmenting compounds were found in the sample of analyzed cosmetic products. A more comprehensive analysis was performed, and the top ten pure depigmenting agents with the highest usage frequency were identified (Figure 2). Hydroxy (HA) and keto acids are the most commonly used depigmenting agents, with 76.1% of the depigmenting products studied having at least one HA or keto acid in their composition. Vitamin C and derivatives were the second most-used depigmenting compounds, totalizing a usage frequency of 62.0%. Similarly, alpha hydroxy acids were found to be the most frequently used depigmenting agents in anti-aging cosmetic products in both 2011 and 2018, while ascorbic acid and its derivatives were ranked as the second most commonly used during the same period [2]. Niacinamide, resorcinol derivatives, peptides and amino acids, azelaic acid and derivatives, tranexamic acid, retinoids, kojic acid, and acetyl glucosamine complete the top ten most-used depigmenting agents in the pool of cosmetic products analyzed, whose usage frequency ranged between 7% and 40%. The usage frequency of HAs and keto acids was notably higher than that of the other nine depigmenting agents. In contrast to previous studies on anti-aging cosmetic products from 2011 and 2018, where retinoids ranked as the third most commonly used depigmenting agent, niacinamide occupies this position in the current analysis, with retinoids appearing at a much lower usage percentage [2]. This difference may be attributed to the fact that retinoids have not only depigmenting properties but also anti-aging effects, which likely contributed to their more frequent use in anti-aging products compared to depigmenting products [12].

Additionally, this study identified a broader range of resorcinol derivatives compared to the anti-aging cosmetic products study [2]. Notably, 4-butylresorcinol was the only resorcinol derivative that appeared among the top most commonly used depigmenting agents in anti-aging products [2]. In another study focusing on both depigmenting cosmetic products and medicines, the most prevalent depigmenting agents identified were kojic acid, arbutin, ascorbic acid, hydroquinone, and glycolic acid, in descending order [6]. In the present study, the same depigmenting agents were found but at lower usage percentages, except for hydroquinone, which was not found since only cosmetic products were analyzed.

**Figure 3:**
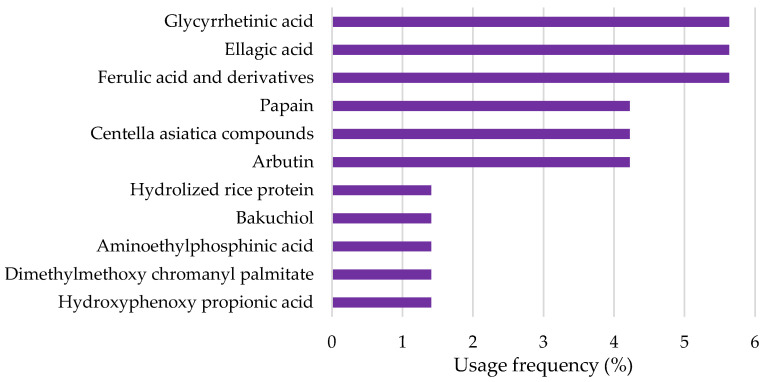
Usage frequency of the remaining depigmenting agents present in the analyzed cosmetic products.

Several other depigmenting agents were identified in the cosmetic product labels, whose frequency of use varied between 1% and 7% (Figure 3). These are mostly natural-derived depigmenting compounds, except for aminoethylphosphinic acid and dimethylmethoxy chromanyl palmitate.

**Figure 4:**
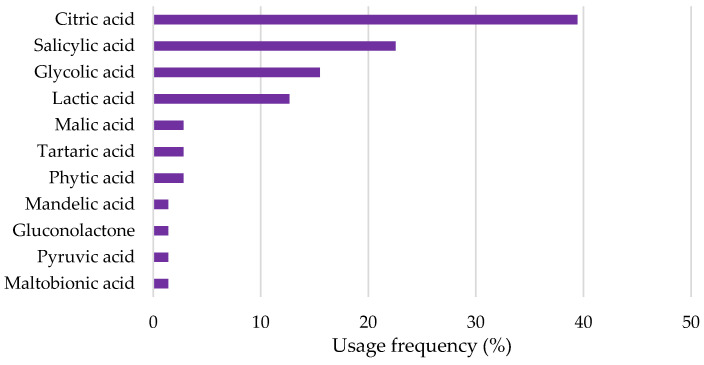
Usage frequency of HAs and keto acids in cosmetic products.

Citric, salicylic, glycolic, and lactic acids were among the most commonly used HAs as depigmenting agents in cosmetic products. The opposite was seen in the malic, tartaric, phytic, mandelic, pyruvic, and maltobionic acids, as well as gluconolactone, that were found in a diminished number among all the formulations analyzed (less than 5%) (Figure 4). Citric acid was used in the highest usage frequency (39.4%), followed by salicylic (22.5%), glycolic (16.9%), and lactic acid (12.7%). The findings align with existing literature, which frequently identifies salicylic acid and glycolic acid as the most commonly used HAs in depigmenting formulations [13,14,15].

**Figure 5:**
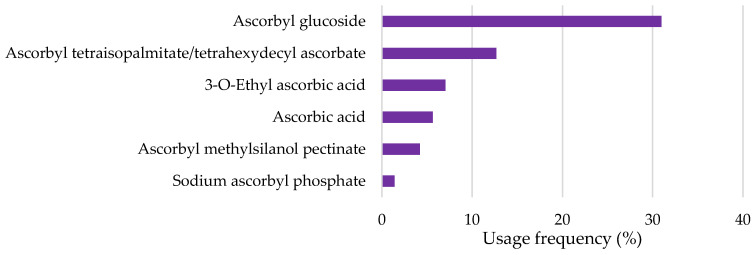
Usage frequency of vitamin C and derivatives in cosmetic products.

Ascorbic acid, mostly known as vitamin C, and its derivatives were also found on the labels of some cosmetic products (Figure 5). Ascorbyl glucoside derivative, with a usage frequency of 31.0%, was notably the most frequent ascorbic acid derivative found on the label of cosmetic formulations, followed by ascorbyl tetraisopalmitate/tetrahexyldecyl ascorbate (12.7%), 3-*O*-ethyl ascorbic acid (7.0%), ascorbic acid (5.6%), and finally ascorbyl methylsilanol pectinate and sodium ascorbyl phosphate, with a usage frequency <5%. The low prevalence of ascorbic acid compared to its derivatives aligns with the existing literature, which describes this compound as highly unstable under both aerobic and anaerobic conditions [16]. In contrast, ascorbyl glucoside was the most frequently identified derivative, likely due to its reported high stability against reduction and oxidation, making it more resistant to degradation [17].

**Figure 6:**
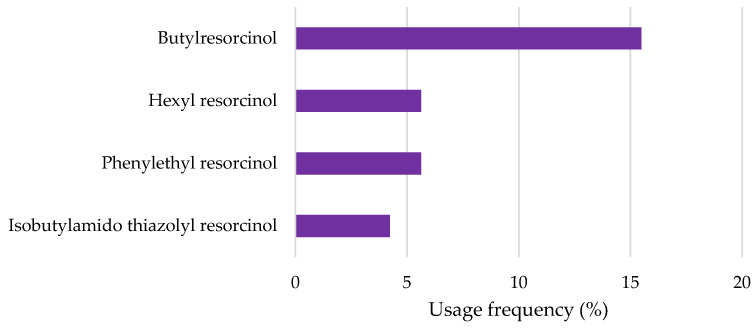
Usage frequency of resorcinol and derivatives in cosmetic products.

Resorcinol derivatives, butylresorcinol, hexyl resorcinol, phenylethyl resorcinol, and isobutylamido thiazolyl resorcinol are also among the top ten most commonly used depigmenting agents in cosmetic formulations (Figure 6). Butylresorcinol is present in 11 of the 70 cosmetic formulations studied, with a usage frequency of 15.5%; hexyl resorcinol and phenylethyl resorcinol were both found in 4 of the 70 total analyzed cosmetic products (5.6%); and isobutylamido thiazolyl resorcinol was the derivative found with the lowest frequency of use (4.2%). Butylresorcinol was the most-used derivative, likely due to its well-documented depigmenting efficacy and extensive research supporting its efficacy [2]. Additionally, its widespread use extends beyond depigmenting formulations, as it is also commonly incorporated into anti-aging cosmetic products [2].

**Figure 7:**
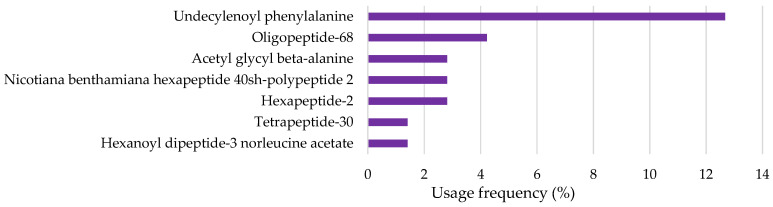
Usage frequency of peptides and amino acids in cosmetic products.

Peptides and amino acids were also found in the analyzed cosmetic formulations, being part of the top ten depigmenting agents (Figure 7). Undecylenoyl phenylalanine (UPhe) was the amino acid most frequently found in cosmetic products (12.7%), followed by oligopeptide-68 (OP68), with a usage frequency of 4.2%, and acetyl glycyl beta-alanine (AGbetaA), nicotiana benthamiana hexapeptide 40sh-polypeptide 2 (NBHP40shPP2), hexapeptide-2 (HP2), tetrapeptide-30 (TP30), and hexanoyl dipeptide-3 norleucine acetate (HDP3NA), with a frequency of use lower than 3%. Peptides and amino acids are among the ten most frequently used depigmenting agents, reflecting their increasing incorporation into cosmetic formulations over the years [18]. This trend is driven by growing research and development efforts due to their favorable biocompatibility and bioactivity, making them attractive candidates for skincare applications [18].

**Figure 8:**
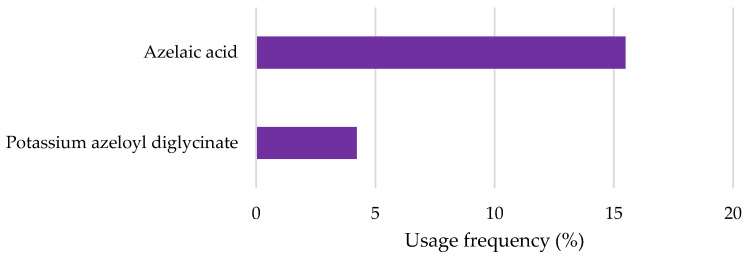
Usage frequency of azelaic acid and derivatives in cosmetic products.

Azelaic acid and its derivative potassium azeloyl diglycinate were found in the sixth position of the top ten depigmenting agents in cosmetic products (Figure 8). Azelaic acid had a markedly higher frequency of use (15.5%) than the derivative (4.2%). The higher usage frequency of azelaic acid may be attributed to its extensive study and established efficacy in the treatment of acne and rosacea [19,20]. Additionally, multiple reports have highlighted its benefits in managing hyperpigmentation, further supporting its widespread incorporation into cosmetic formulations [13].

**Figure 9:**
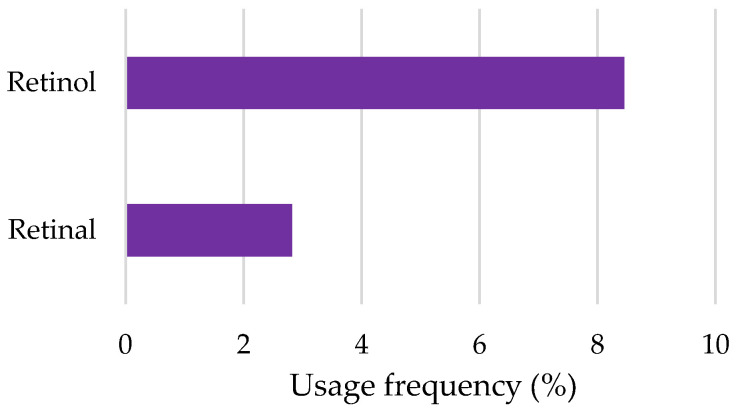
Usage frequency of retinoids in cosmetic products.

Finally, retinoids were among the most frequently used depigmenting agents in cosmetic products (Figure 9). Retinol was the most frequently used depigmenting agent (8.5%), followed by retinal, with a usage frequency of 2.8%. The higher usage frequency of retinol observed in this study is consistent with findings in the literature [2,12]. Compared to retinal, retinol provides effective biological activity while maintaining an adequate skin tolerance [12]. In contrast, retinal is known to be more irritating, which may limit its widespread use in cosmetic formulations [12].

### 2.2. Scientific Evidence Regarding Aquatic Toxicity and Biodegradability of Most-Used Depigmenting Agents

#### 2.2.1. Hydroxy (HA) and Keto Acids

**Figure 10:**
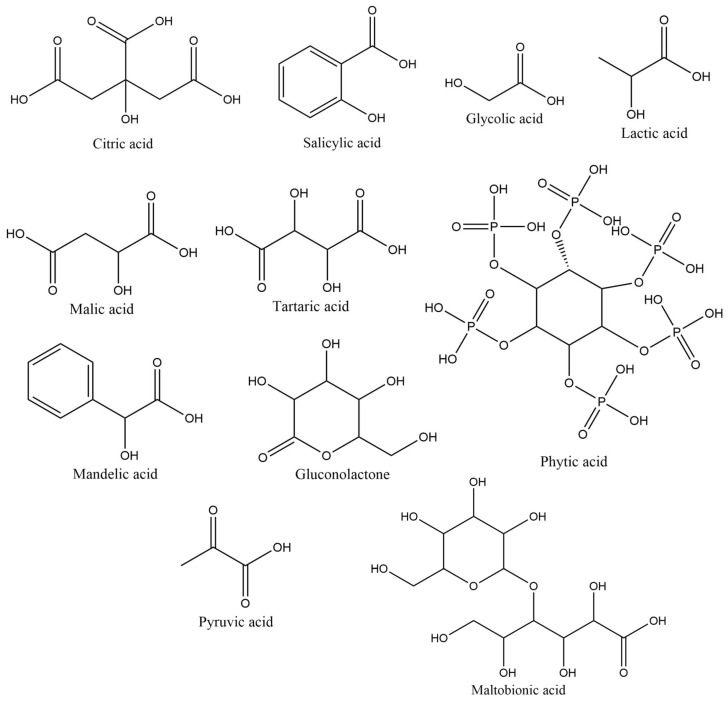
Chemical structure of the HAs and keto acids identified in the analyzed cosmetic products.

Hydroxy acids (HAs) are a group of naturally derived compounds, which can also be synthesized and extensively used in several cosmetic and dermatological formulations to provide multiple skin benefits, particularly for improving photoaged skin [21]. In cosmetic formulations, these compounds are typically used at concentrations up to 10%, which suggests that their overall environmental impact is considered low [22]. HAs specifically target hyperkeratinization by causing a rapid detachment of the innermost layer of the hyperkeratotic stratum corneum [23,24]. Their efficacy is evidenced by the reduction in skin roughness, discoloration, solar keratoses, and overall pigmentation, along with enhancements in collagen density and the quality of elastic fibers [21,25]. The antiaging and depigmenting properties of HAs have established them as key ingredients in cosmetic products that function as exfoliants and moisturizers. There are four categories of HAs: alpha-hydroxy acids (AHAs), beta-hydroxy acids (BHAs), polyhydroxy acids (PHAs), and bionic acids (BAs). Figure 10 shows the chemical structure of HAs and keto acids found in the cosmetic products analyzed. AHAs are a class of carboxylic acids characterized by a hydroxyl group located at the alpha position relative to the carboxyl group [21,23]. The hydroxyl and carboxyl groups are attached directly to an aliphatic carbon, with the hydroxyl group being neutral, while the acidic characteristic is solely attributed to the carboxyl group [23]. Due to their hydrophilic nature, AHAs primarily exhibit their effects on the skin’s surface by exfoliation of the stratum corneum [26]. This mechanism promotes skin renewal and is associated with moisturizing and anti-aging effects such as improved texture and reduction of fine lines [26]. Glycolic acid, derived from sugar cane, is the smallest and simplest AHA. Due to its small molecular size and thus superior ability to penetrate the skin, it is the most widely used AHA in cosmetic formulations [21,23,27]. Lactic acid is another AHA, derived from milk, and is commonly used in topical formulations due to its hydrating, exfoliating, and anti-aging properties [21,23,27]. Phytic acid and mandelic acid are also classified as AHAs [27,28]. Mandelic acid, derived from almonds, has a relatively larger molecular structure, which allows for slower skin penetration, and, consequently, the risk of irritation is minimized [27].

Beta-hydroxy acids (BHAs) are a type of carboxylic acid characterized by the presence of a hydroxyl group attached to the beta-position relative to the carboxyl group [21,23]. Similar to AHAs, the hydroxyl group in BHAs is neutral, while the carboxyl group confers acidity [23]. The most common example of a BHA is beta-hydroxy butanoic acid [23]. Although salicylic acid is often referred to as a BHA, this classification is technically inaccurate [23]. In salicylic acid, both the hydroxyl and carboxyl groups are directly bonded to an aromatic benzene ring, and each one contributes to its acidic properties [23,29]. Salicylic acid is lipophilic, unlike AHAs, and thus has a strong affinity for epidermal lipids and sebaceous gland lipids found in hair follicles [29,30]. This property gives salicylic acid its keratolytic and comedolytic effects [29,30]. Citric acid, malic acid, and tartaric acid are considered both AHAs and BHAs due to the presence of hydroxyl groups in the alpha-position to one carboxyl group and in the beta-position to another; however, these primarily function as AHAs [21,23,31,32]. Both AHAs and BHAs act as skin exfoliants; however, BHAs can improve overall skin texture without irritating, unlike AHAs. Polyhydroxy acids (PHAs) are a class of carboxylic acids characterized by the presence of two or more hydroxyl groups bonded to the carbon atoms of an aliphatic chain [23]. The acidity of PHAs is solely due to their carboxyl group, while the hydroxyl groups remain neutral [23]. Among PHAs, gluconolactone is frequently used in cosmetic formulations due to its accessibility and its ability to provide anti-aging effects similar to those of HAs [21,23,33]. Bionic acids (BAs) are categorized as aldobionic acids, which are composed of a carbohydrate monomer covalently bonded to an aldonic acid [23]. Examples of BAs include lactobionic acid, maltobionic acid, and cellobionic acid [23]. Typically, BAs are derived from their corresponding disaccharides via chemical or enzymatic oxidation processes [23]. Both PHAs and BAs provide skin benefits similar to HAs although with a lower risk of irritation, as their larger molecular size limits their ability to penetrate deeper layers of the skin [34].

Pyruvic acid is classified as a naturally derived alpha-keto acid (AKA) since it possesses a ketone group at the alpha-position relative to its carboxyl group [32]. Structurally, AKAs, including pyruvic acid, resemble AHAs but differ in having a ketone group in place of a hydroxyl group at the alpha-position. Although pyruvic acid itself is not an AHA, it can be converted into lactic acid, an AHA, in the skin via the enzyme lactic dehydrogenase [35]. This enzymatic conversion allows AKAs like pyruvic acid to exhibit similarities to AHAs in cosmetic applications [35]. Additionally, pyruvic acid has been shown to have keratolytic, antimicrobial, and sebum-regulating properties [36,37].

Data on the aquatic toxicity of HAs and keto acids remains limited. Table 1 summarizes the data on the acute aquatic toxicity and biodegradability of each HA and keto acid found in the cosmetic products analyzed. The biodegradability data showed that all tested HAs and keto acids are readily biodegradable. Additionally, none of the compounds pose a risk of acute aquatic toxicity, as their LC_50_ or EC_50_ values surpass 100 mg/L [38].

#### 2.2.2. Vitamin C and Derivatives

**Figure 11:**
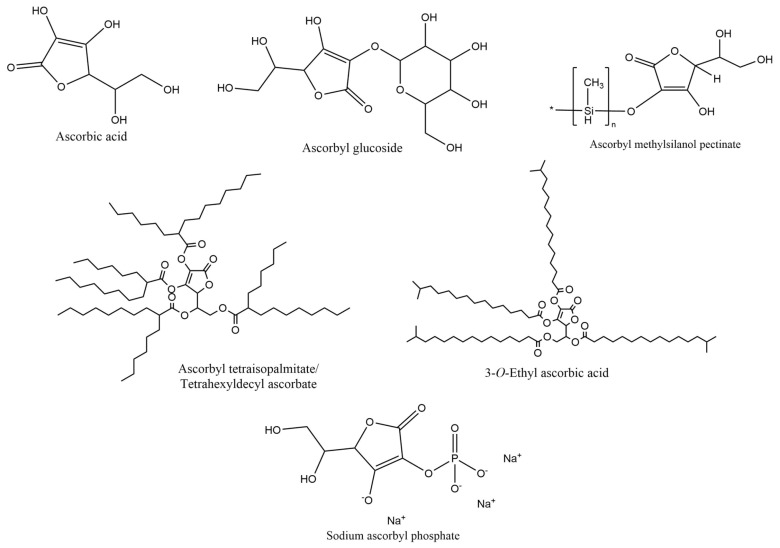
Chemical structure of the vitamin C and derivatives identified in the analyzed cosmetic products.

Vitamin C, also known as ascorbic acid, is a water-soluble compound that exists in nature in two forms: the reduced form (L-ascorbic acid or ascorbate) and the oxidized form (L-dehydroascorbic acid) [16,50]. In cosmetic applications, vitamin C acts as an antioxidant by neutralizing reactive oxygen species (ROS) [16,50]. It has also shown efficacy in improving conditions such as hyperpigmentation, melasma, and sunspots, which stem from the capacity of vitamin C to interact with the active site of tyrosinase, the enzyme that regulates melanogenesis [16,51]. Additionally, vitamin C promotes keratinocyte differentiation and enhances the cohesion of the dermal–epidermal junction [16,52]. However, ascorbic acid is notoriously unstable, degrading rapidly in aqueous solutions, under alkaline pH conditions, and in the presence of oxygen, light, and metal ions [53]. To enhance its stability, various chemical modifications have been developed, resulting in both hydrophilic (ascorbyl glucoside, ascorbyl methylsilanol pectinate, and sodium ascorbyl phosphate) and lipophilic (ascorbyl tetraisopalmitate/tetrahexyldecyl ascorbate and 3-*O*-ethyl ascorbic acid) derivatives of vitamin C that can be also found in cosmetic products [54]. In cosmetic formulations, vitamin C and its derivatives are commonly incorporated at concentrations of up to 10%. Figure 11 shows the chemical structure of vitamin C and its derivatives found in the cosmetic products analyzed.

Research on the aquatic toxicity of vitamin C and its derivatives is still scarce. According to a study conducted by the OECD, L-ascorbic acid is naturally synthesized within organisms and thus is characterized by low toxicity to environmental organisms [55]. Furthermore, the impact of industrial production and emissions of L-ascorbic acid on environmental ecosystems is anticipated to be minimal, as these activities contribute only a small portion of the total L-ascorbic acid found in the environment [55]. Table 2 summarizes the data on the acute aquatic toxicity and biodegradability of vitamin C and each derivative found in the cosmetic products analyzed. For the ascorbyl methylsilanol pectinate derivative, no information is currently available regarding its biodegradability or aquatic toxicity. Most derivatives were not considered readily biodegradable, only ascorbic acid and ascorbyl glucoside derivatives. For these compounds, the risk of acute aquatic toxicity is also low considering the LC_50_ and EC_50_ values were close to or above 100 mg/L. Therefore, ascorbic acid and the ascorbyl glucoside derivative present a low environmental risk. Ascorbyl tetraisopalmitate/tetrahexyldecyl ascorbate presented the greatest risk of acute aquatic toxicity since it is not readily biodegradable, and the LC_50_ and EC_50_ values are quite low (<1 mg/L). Additionally, this derivative exhibits an oil–water partition coefficient (log P) greater than 6.2, indicating high lipophilicity and, consequently, a strong affinity for lipids, which may facilitate their accumulation in the fatty tissues of aquatic organisms [56]. Therefore, ascorbyl tetraisopalmitate/tetrahexyldecyl ascorbate may have a greater environmental risk. 3-*O*-Ethyl ascorbic acid and sodium ascorbyl phosphate, despite not being readily biodegradable, present a low risk of acute aquatic toxicity (LC_50_ and EC_50_ close to or above 100 mg/L). 3-*O*-Ethyl ascorbic acid has a log P greater than −0.8, and sodium ascorbyl phosphate has a log P greater than −4.5, indicating reduced lipid affinity and, consequently, a low risk of accumulation in fatty tissues within organisms [57,58].

#### 2.2.3. Niacinamide

**Figure 12:**
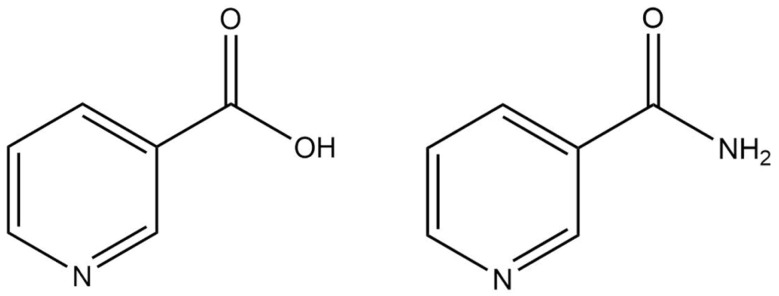
Chemical structure of niacin (**left**) and niacinamide (**right**).

Niacinamide, also referred to as nicotinamide or vitamin B3, is the biologically active form of niacin, which can be obtained from natural or synthetic sources (Figure 12) [61,62]. It exhibits several potential benefits for the skin, including anti-inflammatory and antioxidant properties [61,62,63,64]. Additionally, it promotes intercellular lipid production and serves as an effective skin depigmenting agent by blocking the transfer of melanosomes from melanocytes to keratinocytes [61,62,63]. Niacinamide is typically included in cosmetic formulations, and these compounds are commonly incorporated into products at concentrations of up to 3% [65].

Data regarding the environmental effects of niacinamide remain limited. ECHA classifies niacinamide as readily biodegradable based on results from the Modified OECD Screening Test, following OECD Guideline 301 E [66]. For acute aquatic toxicity, the following results were observed: mortality of *Poecilia reticulata* (tested according to EU Method C.1 and OECD Guideline 203) showed an LC_50_ > 1000 mg/L after 96 h; immobilization of *Daphnia magna* (tested following EU Method C.2 and OECD Guideline 202) resulted in an EC_50_ > 1000 mg/L after 24 h; and inhibition of algal growth (evaluated using EU Method C.3 and OECD Guideline 201) produced an IC_50_ > 1000 mg/L after 72 h [66]. Based on the gathered data, niacinamide poses no significant environmental threat, as it is a biodegradable compound with a low risk of acute aquatic toxicity (LC_50_, EC_50_, or IC_50_ > 100 mg/L) [38].

#### 2.2.4. Resorcinol Derivatives

**Figure 13:**
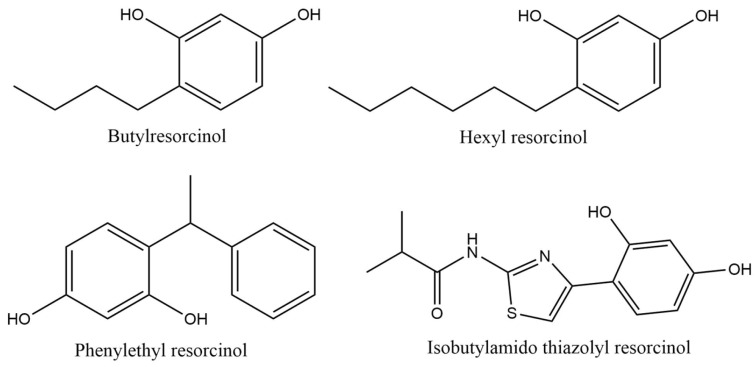
Chemical structure of the resorcinol derivatives identified in the analyzed cosmetic products.

Resorcinol derivatives are the most-used tyrosinase inhibitors in depigmenting cosmetic products [2,67]. These resorcinol derivatives are synthetically derived and are commonly incorporated into cosmetic formulations at concentrations not exceeding 4%. The resorcinol moiety is well known for its role in tyrosinase inhibition, specifically C_4_ alkyl-substituted resorcinol derivatives, which were found in the cosmetic products examined (Figure 13).

Phenol and its derivatives are considered significant environmental contaminants mainly due to their occurrence in several industrial wastewaters, being highly toxic to aquatic organisms [68]. In fact, resorcinol itself is an environmental pollutant that can bioaccumulate in organisms, potentially disrupting endocrine functions and altering systemic biological processes [69,70]. Likewise, computational and in vitro studies of biodegradation in water and acute aquatic toxicity were carried out for resorcinol derivatives (Table 3). Regarding biodegradability, the results show that none of the resorcinol derivatives are readily biodegradable. Butylresorcinol proved to be the derivative with the highest acute aquatic toxicity, with the lowest EC_50_ value for the immobilization test of *Daphnia magna*. The derivative isobutylamido thiazolyl resorcinol showed the highest algae toxicity, with the lowest EC_50_ value for the algal growth and biomass inhibition test of *Desmodesmus subspicatus*. Toxicity to fish, through the evaluation of the mortality of *Danio rerio*, and to microorganisms, through the inhibition of total respiration in activated sludge, has only been tested for phenylethyl resorcinol. The log P values of these resorcinol derivatives range from 2 to 4, suggesting a potential affinity for lipids and, consequently, for accumulation in the fatty tissues of organisms [71,72,73,74]. Overall, resorcinol derivatives pose a potential environmental hazard due to their lack of biodegradability and ability to cause acute aquatic toxicity at concentrations below 100 mg/L [38].

#### 2.2.5. Peptides and Amino Acids

Amino acids are the fundamental components of peptides and proteins, characterized by a carbon skeleton with at least one amino group and one carboxyl group [75,76]. Peptides, which are smaller than proteins, consist of short chains of typically two to about fifty amino acids linked via peptide bonds [75,77]. Bioactive peptides, such as collagen hydrolysate and collagen peptides derived from various sources, have shown beneficial effects on the skin, including antioxidant, anti-aging, moisturizing, and collagen-stimulating properties [75,78,79,80,81,82]. The activity of these bioactive peptides is ultimately determined by their molecular weight, hydrophobicity, and amino acid sequence [78]. Additionally, some amino acids and peptides contribute to skin depigmentation. This effect can occur through mechanisms such as inhibiting the active site or chelating copper ions of tyrosinase, suppressing the activation of the microphthalmia-associated transcription factor (MITF), a key regulator in melanogenesis, or downregulating the cAMP signaling pathway, which plays a role in anti-melanogenic activity [75,78,83,84,85,86]. The analysis of the depigmenting products revealed the presence of only one amino acid: undecylenoyl phenylalanine (UPhe), which is of synthetic origin. Additionally, several peptides were identified, including oligopeptide-68 (OP68), acetyl glycyl beta-alanine (AGbetaA), nicotiana benthamiana hexapeptide 40 SH-polypeptide 2 (NBHP40shPP2), hexapeptide-2 (HP2), tetrapeptide-30 (TP30), and hexanoyl dipeptide-3 norleucine acetate (HDP3NA). Among these peptides, only NBHP40shPP2 is of natural origin, while the others are synthetic and specifically designed to reduce hyperpigmentation. Peptides and amino acids are typically included in cosmetic formulations at concentrations up to 5%.

Data regarding the environmental effects of peptides and amino acids are limited. Table 4 presents a summary of the acute aquatic toxicity and biodegradability data for the various peptides and amino acids identified in the analyzed cosmetic products. Currently, there is a lack of data regarding the analyzed parameters for the peptides NBHP40shPP2, HP2, and HDP3NA. Among the compounds, UPhe and AGbetaA are identified as readily biodegradable, whereas OP68 and TP30 have been predicted to be not readily biodegradable based on computational analyses. The log P values of OP68 (−6.8) and TP30 (−8.3) indicate minimal lipid affinity, suggesting a low potential for accumulation in the fatty tissues of organisms. In terms of acute aquatic toxicity, only UPhe and AGbetaA have documented information, and both demonstrate a low toxicity risk, with EC_50_ values close to or above 100 mg/L. The information collected regarding UPhe and AGbetaA indicates that they pose minimal environmental risk.

#### 2.2.6. Azelaic Acid and Derivatives

Azelaic acid is a naturally occurring dicarboxylic acid found in grains such as barley, wheat, and rye [89]. Additionally, it is synthesized by *Malassezia furfur*, a yeast responsible for the skin disorder known as Pityriasis versicolor, which disrupts the process of melanogenesis and leads to hypopigmentation [89,90]. This compound exhibits inhibitory effects on tyrosinase and, to an even greater degree, on thioredoxin, contributing to skin depigmentation [91,92,93]. The thioredoxin enzyme contributes to melanogenesis by modulating tyrosinase activity through a feedback mechanism [91,93]. This process involves the transfer of electrons to intracellular thioredoxin, modulating cellular redox signaling pathways that can regulate tyrosinase [91,93]. Furthermore, azelaic acid possesses a broad range of skin-beneficial properties, including antibacterial and anti-inflammatory properties and the ability to reduce excessive keratinization [91,94]. Nonetheless, azelaic acid presents formulation challenges, primarily because effective concentrations are required for its skin effects [95]. At high concentrations, it exhibits limited solubility and negatively affects the cosmetic attributes of formulations, particularly in terms of spreadability [95]. Azelaic acid and its derivatives are commonly incorporated into cosmetic formulations at concentrations of up to 10%.

**Figure 14:**
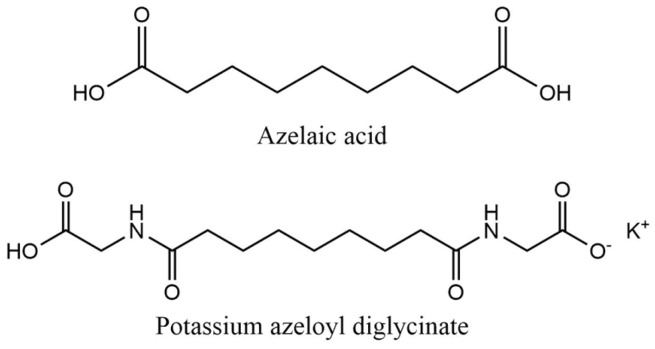
Chemical structure of the azelaic acid and its derivatives identified in the analyzed cosmetic products.

This has led to the synthetic development of potassium azeloyl diglycinate, a soluble form of azelaic acid that retains the cosmetic benefits of azelaic acid while enhancing its technical properties, including solubility and skin compatibility [95]. Figure 14 shows the chemical structure of azelaic acid and its derivatives found in the cosmetic products analyzed.

There is a lack of information available regarding environmental data on azelaic acid and its derivatives. Table 5 provides a concise overview of the acute aquatic toxicity and biodegradability profiles of azelaic acid and its derivatives identified in the analyzed cosmetic products. Azelaic acid and its derivative, potassium azeloyl diglycinate, exhibit ready biodegradability. Additionally, azelaic acid possesses a higher potential for acute aquatic toxicity, indicated by an LC_50_ and EC_50_ < 100 mg/L. Nevertheless, due to the biodegradable nature of azelaic acid, the associated environmental risks are expected to be reduced.

#### 2.2.7. Tranexamic Acid

**Figure 15:**
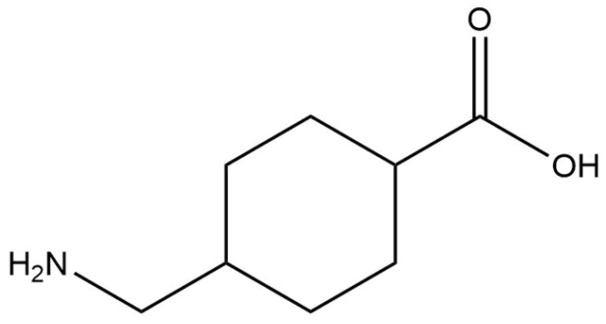
Chemical structure of tranexamic acid.

Tranexamic acid is a synthetic derivative of the amino acid lysine and functions as a plasmin inhibitor, preventing fibrinolysis (Figure 15) [99]. Its mechanism of action specifically involves blocking the conversion of plasminogen to plasmin through the inhibition of the plasminogen activator [99,100,101]. Exposure to UV radiation stimulates the production of this activator, leading to increased plasmin activity in keratinocytes, release of intracellular arachidonic acid, and elevated levels of alpha-melanocyte-stimulating hormone [99,100,102,103]. Both plasmin and alpha-melanocyte-stimulating hormone promote melanin synthesis [99,100,103]. Consequently, tranexamic acid’s ability to inhibit plasmin is considered a key factor in its skin depigmentation effects. Additionally, due to its structural similarity to tyrosine, tranexamic acid can competitively inhibit the activity of tyrosinase [99,100,104]. Tranexamic acid is commonly incorporated into cosmetic formulations up to 5%. There is limited information concerning the environmental data of tranexamic acid. According to ECHA, tranexamic acid is classified as readily biodegradable. The substance was evaluated for biodegradability using the manometric respirometry test following OECD Guideline 301 F, achieving an 81.94% degradation rate over 28 days, indicating it is readily biodegradable [105]. Acute aquatic toxicity was assessed through the immobilization of *Daphnia magna* following OECD Guideline 202, resulting in a 48 h EC_50_ value exceeding 100 mg/L, suggesting low acute toxicity to aquatic invertebrates. Similarly, algal growth inhibition, measured using OECD Guideline 201, resulted in an EC_50_ value exceeding 100 mg/L following 72 h of exposure [105]. The data collected showed that tranexamic acid does not display a significant environmental hazard, as it is readily biodegradable and exhibits a low potential for acute aquatic toxicity (EC_50_ > 100 mg/L).

#### 2.2.8. Retinoids

**Figure 16:**
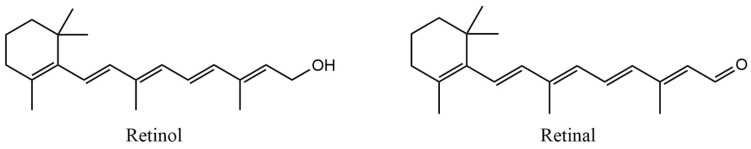
Chemical structure of retinol and its derivatives identified in the analyzed cosmetic products.

Retinol, retinal, and retinoic acid are classified as retinoids, a group of compounds characterized by an isoprenoid chain linked to a beta-ionone ring and which can be found in both natural forms and synthetic derivatives (Figure 16) [106,107]. Retinoids are commonly used as skin anti-aging agents in cosmetic products due to their strong affinity for retinoic acid receptors (RARs) and retinoid X receptors (RXRs) in the nucleus [106,108,109,110]. These interactions stimulate keratinocyte proliferation, enhance the epidermis’ barrier function, reduce transepidermal water loss, protect collagen from breakdown, and inhibit the action of metalloproteinases [107,110]. These retinoids are typically incorporated into cosmetic formulations at concentrations of up to 1%.

Retinol (all-trans retinol), also known as vitamin A, is a retinoid that remains stable in cosmetic formulations and is generally well tolerated by the skin [107,111]. It undergoes a two-step oxidation process to be converted into its active form, retinoic acid [108]. Initially, retinol is oxidized to retinaldehyde (retinal), which is further oxidized irreversibly into retinoic acid, the biologically active compound [108]. On the other hand, retinol can also interact with retinoic acid receptors [107]. Retinol is recognized for its ability to enhance skin texture, address depigmentation, reduce dryness, and minimize the appearance of fine lines [106,107,112].

Retinal, or retinaldehyde, is the aldehyde derivative of retinoic acid and represents the oxidized form of retinol [107,113]. While retinal is incorporated into cosmetic formulations, its effectiveness remains limited [107,113]. As a more stable and less irritating alternative to retinoic acid, it provides modest improvements in wrinkle reduction and skin texture [107,113]. Additionally, retinal is used in the improvement of photoaging skin signs [106,113].

Retinoids have been found in aquatic environments such as seawater, rivers, lakes, and sewage treatment plants [114]. The toxicity of retinoids to aquatic organisms is also well documented. Table 6 provides a summary of data on acute aquatic toxicity and biodegradability for the retinoids identified in the cosmetic products analyzed in this study. Retinol was found to be readily biodegradable, unlike retinal. Retinol showed a low potential for aquatic toxicity, as LC_50_ and EC_50_ values observed for the aquatic species studied exceeded 100 mg/L. This suggests that retinol poses a minimal environmental hazard due to its biodegradability and limited toxicity in aquatic environments. However, retinal is a degradation/oxidation product of retinol, and it is no longer considered readily biodegradable, possibly exhibiting greater toxicity to aquatic systems. Thus, studying both the degradation products and their toxicity alongside the parent compound is crucial. No data were available on the aquatic toxicity of retinal, however, its log P value of 6.2 indicates a strong affinity for lipids, suggesting that it can greatly accumulate in the fatty tissues of aquatic organisms [115].

#### 2.2.9. Kojic Acid

**Figure 17:**
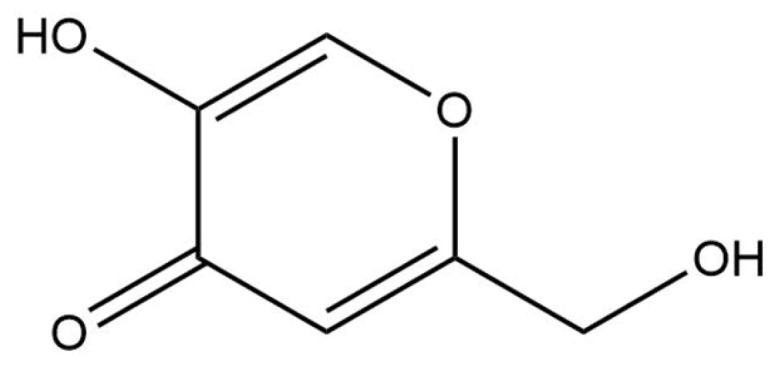
Chemical structure of kojic acid.

Kojic acid is a commonly used skin depigmenting agent in cosmetic formulations (Figure 17). It is produced by various fungi, including *Aspergillus flavus*, *Aspergillus oryzae*, *Aspergillus tamarii*, and *Aspergillus parasiticus*, through an aerobic fermentation process [117,118]. Additionally, kojic acid is a by-product of the fermentation of certain traditional Asian foods, such as soy sauce and sake [117,118,119]. Kojic acid functions as a slow, reversible tyrosinase inhibitor by chelating the copper ion in the enzyme’s active site, thereby inhibiting melanin production, which is responsible for skin pigmentation [117,119,120]. However, kojic acid is associated with stability and safety concerns, potentially causing adverse skin effects [119]. As a result, ongoing research focuses on developing kojic acid analogues with improved efficacy for treating hyperpigmentation, along with enhanced stability and safety profiles [119]. Kojic acid is commonly included in cosmetic formulations at concentrations of up to 2%.

The data on the environmental impact of kojic acid are scarce, especially in terms of biodegradability and toxicity in aquatic environments. In an in silico assessment using BioWin v4.10, kojic acid was predicted to be readily biodegradable. Additionally, aquatic toxicity predictions using ECOSAR v1.11 indicated that the 48 h LC_50_ for *Daphnia* species is approximately 0.90 mg/L; for green algae, the 96 h LC_50_ is 1666.56 mg/L; and for fish, the 96 h LC_50_ is estimated at 8.44 mg/L. Although kojic acid exhibits a low LC_50_ for *Daphnia magna* and fish, its ready biodegradability suggests that the overall risk of aquatic toxicity remains relatively low.

#### 2.2.10. *N*-Acetylglucosamine

**Figure 18:**
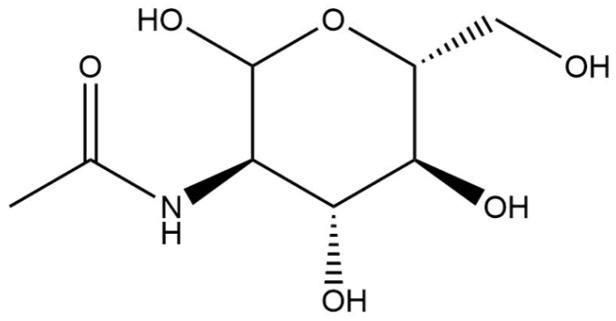
Chemical structure of *N*-acetylglucosamine.

*N*-Acetylglucosamine, a monosaccharide derived from glucose, features a linear polymer structure characterized by (1,4)-beta-linkages (Figure 18) [121]. It is an integral component of several heterogeneous polysaccharides, including hyaluronic acid, which is found in the skin [121]. Hyaluronic acid is synthesized primarily by fibroblasts and keratinocytes, playing a crucial role in retaining moisture within the stratum corneum and dermis [121]. The use of *N*-acetylglucosamine on the skin has been shown to stimulate the proliferation of both keratinocytes and fibroblasts, thereby enhancing hyaluronic acid production and improving skin hydration and wrinkles [121,122,123]. Additionally, beyond its moisturizing properties, *N*-acetylglucosamine has shown efficacy in reducing skin hyperpigmentation [124,125]. Its depigmenting effects arise from its ability to decrease melanin synthesis through the upregulation of several genes associated with epidermal turnover and antioxidant responses, alongside the downregulation of cytoskeletal genes that facilitate melanosome transport [121]. *N*-Acetylglucosamine is frequently incorporated into cosmetic formulations at concentrations reaching up to 5%.

Data regarding the biodegradability and aquatic toxicity of *N*-acetylglucosamine are limited. *N*-Acetylglucosamine is considered readily biodegradable, achieving 80.1% degradation by the conclusion of a 28-day study conducted using the Closed Bottle Test method outlined in OECD Guideline 301 D [126]. In terms of aquatic toxicity, QSAR predictions revealed that the LC_50_ for fish mortality after 96 h of exposure is 13,575.4 mg/L, while for *Daphnia* sp. mortality after 48 h, the LC_50_ is estimated at 22,624.07 mg/L [126]. Additionally, the EC_50_ for the inhibition of green algae growth after 96 h of exposure was 6596.19 mg/L [126]. Considering the biodegradable nature of *N*-acetylglucosamine and its relatively high LC_50_/EC_50_ values concerning aquatic toxicity, it can be inferred that this compound poses a minimal risk to the environment.

## 3. Materials and Methods

### 3.1. Data Collection

A market analysis was conducted to evaluate the composition of cosmetic products regarding the presence of depigmenting ingredients. The label information of a pool of 70 depigmenting cosmetic products from 23 international cosmetic brands marketed in Portuguese parapharmacies and pharmacies was collected in 2022 to access the presence of skin depigmenting agents.

#### 3.1.1. Product Selection Criteria

The selection of products was based on specific inclusion criteria. Only products that mentioned skin depigmenting claims on their labels were included. These claims were identified using specific terms such as “spot”, “whitening”, and “depigmenting”.

#### 3.1.2. Data Sources

All available information on the product’s label, as well as additional data from manufacturers’ websites, was compiled.

#### 3.1.3. Exclusion Criteria

This study was limited to cosmetic products that exclusively contained pure compounds with recognized skin depigmenting activity. Complex mixtures of compounds such as natural extracts were excluded from the analysis.

#### 3.1.4. Ecotoxicity Analysis

After identifying the most-used depigmenting agents, an analysis of putative ecotoxicity was performed for these compounds. This analysis was based on scientific data available in the literature regarding the environmental risks of the depigmenting agents.

### 3.2. Data Analysis

#### 3.2.1. Ingredient Identification and Classification

The depigmenting agents in the cosmetic products were listed according to the International Nomenclature of Cosmetic Ingredient (INCI).

#### 3.2.2. Analysis Parameters

- Top Depigmenting Agents Used in Cosmetic Products

The depigmenting agents were identified from INCI lists, as previously described, and ranked in descending order of occurrence to disclose the top ten most-used depigmenting agents in cosmetic products.

- Scientific Evidence Regarding Aquatic Toxicity and Biodegradability of Depigmenting Agents

The scientific evidence for each depigmenting ingredient was gathered from the online scientific databases Scopus, ECOTOX, PubChem, and Google Scholar, as well as the registration dossiers/scientific reports of the European Chemicals Agency (ECHA). The keywords used in the search of the online databases included the INCI or IUPAC names of the depigmenting ingredients combined with the terms “ecotoxicity”, “biodegradability”, “aquatic toxicity”, “environmental toxicity”, and “environmental impact”. In cases where the literature data were unavailable, in silico studies were performed by the group to assess biodegradability using a quantitative structure–activity relationship (QSAR) model prediction through BioWin v4.10 software.

Aquatic toxicity tests use several types of organisms to evaluate the effects of chemical substances across different trophic levels. Some of the most commonly used are *Danio rerio* (zebrafish), due to its sensitivity to pollutants and ease of laboratory maintenance; *Daphnia magna*, a freshwater crustacean, widely employed for its rapid reproduction and high sensitivity to contaminants; microalgae, such as *Pseudokirchneriella subcapitata*, as indicators of chemical impact on photosynthesis and growth; and bioluminescent bacteria like *Vibrio fischeri,* to assess toxicity by measuring changes in light emission, providing insights into chemical effects in aquatic environments [127].

## 4. Conclusions

The skin depigmenting sector is undergoing significant growth within the cosmetics industry, driven by rising consumer demand for products that promote an even skin tone. Concurrently, consumer awareness regarding environmental issues is increasing, prompting a shift toward products that are less harmful to the environment. As the use of depigmenting cosmetic products becomes more widespread, it is important to identify the most commonly used depigmenting agents and to conduct a comprehensive assessment of their potential environmental impact, with particular attention to their biodegradability as well as aquatic occurrence and toxicity. This study provides a current overview of the most frequently used depigmenting compounds across 70 commercial products from international brands available on the Portuguese market, along with a compilation of the scientific evidence concerning the biodegradability and aquatic toxicity of the most-used depigmenting agents.

A total of 58 distinct depigmenting compounds were identified, and a list of the ten most frequently used agents was compiled. The HAs and keto acids (76.1%) and vitamin C and derivatives (62.0%) were the most-used depigmenting agents. The remaining top ten depigmenting agents, with usage frequencies ranging from 7% to 40%, included niacinamide, resorcinol derivatives, peptides and amino acids, azelaic acid and its derivatives, tranexamic acid, retinoids, kojic acid, and acetyl glucosamine. The depigmenting agents identified as presenting the highest environmental risk due to their non-biodegradable nature and high aquatic toxicity include the vitamin C derivative ascorbyl tetraisopalmitate/tetrahexyldecyl ascorbate as well as the resorcinol derivatives. The majority of these depigmenting agents are synthetic, while the remaining top ten ingredients are naturally occurring. Natural compounds are likely to pose a lower environmental risk than their synthetic counterparts. Additionally, among the non-readily biodegradable compounds, the majority exhibited high lipophilicity, which increases their potential for bioaccumulation in aquatic organisms and, consequently, poses a higher environmental risk, in contrast to compounds with lower lipophilicity or those that are water-soluble. Therefore, the findings suggest that synthetic and lipophilic depigmenting ingredients pose a higher environmental risk and, consequently, warrant closer monitorization.

Furthermore, studies into the potential risks of metabolites generated during biodegradation are important, as these products may exhibit varying levels of environmental toxicity. Compounds should biodegrade into non-toxic, non-bioaccumulative by-products. For instance, while retinol is readily biodegradable and exhibits low aquatic toxicity, its oxidation produces retinal, a compound no longer considered readily biodegradable and potentially toxic. Adjusting the molecular structure of the synthetic ingredients can improve their biodegradability, reduce the production of harmful by-products, and decrease their persistence in ecosystems. Sourcing from renewable sources is also a strategy that could be pursued.

Both biodegradation and aquatic toxicity studies, as well as the physicochemical properties of chemical substances, serve as key indicators for predicting environmental fates and potential risks for aquatic ecosystems. This study compiles valuable preliminary evidence to guide future research on the environmental safety of these ingredients. The insights obtained in this study underscore the need for regulatory measures that consider the environmental impact of cosmetic ingredients. In fact, the Green Deal and the EU Chemicals Strategy for Sustainability have initiated a focused revision of the EU Cosmetics Regulation, aimed at improving protection for both human health and the environment in the context of cosmetic product usage. Future research should also focus on evaluating the occurrence of the studied depigmenting agents in aquatic systems, as well as expanding the environmental risk assessment studies to include other frequently used cosmetic ingredients. Additionally, further studies could explore the potential chronic/genotoxic effects of these compounds in aquatic organisms. Potential strategies to mitigate the environmental risks of these compounds should be explored, like molecular modifications aimed at reducing their environmental toxicity while preserving their depigmenting effectiveness and ensuring skin safety through a safe and sustainable by design approach. This study enhances knowledge on commonly used depigmenting agents in health product markets and their chemical structures, highlighting potential for innovation in the chemical and pharmaceutical industries as these findings underscore the importance of developing safer and more sustainable alternatives.

## Figures and Tables

**Table 1:** Overview of biodegradability and acute aquatic toxicity data for the HAs and keto acids identified in the analyzed cosmetic products.

Hydroxy Acid/Keto Acid	Biodegradation in Water	Aquatic Toxicity	Reference
**Citric acid**	Modified OECD Guideline 301E Screening Test: 100% degradation in 19 days Readily biodegradable	Immobilization of *Daphnia magna* (OECD Guideline 202): EC_50_ = 1535 mg/L after 24 h	[39]
**Salicylic acid**	QSAR prediction (BioWin v4.10): Readily biodegradable	Immobilization of *Daphnia magna* (OECD Guideline 202): EC_50_ = 2870 mg/L after 48 h Algae growth inhibition test (OECD guideline 201): EC_50_ > 100 mg/L after 72 h Inhibition of total respiration in activated sludge: IC_50_ > 1000 mg/L after 3 h	[40]
**Glycolic acid**	Closed Bottle Test: Degradation after 7 days was 89.6% Readily biodegradable	Mortality of *Danio rerio* (zebra fish): LC_50_ > 5000 mg/L No mortality was observed	[41]
**Lactic acid**	QSAR prediction: Probability of ready biodegradability = 0.936 Readily biodegradable	Mortality of *Lepomis macrochirus:* LC_50_ = 130 mg/L 96 h Immobilization of *Daphnia magna* (OECD Guideline 202): EC_50_ = 130 mg/l after 48 h EC_50_ ≥ 2.8 g/L after 72 h Inhibition of total respiration in activated sludge: NOEC ≥ 88.2 mg/L	[42]
**Malic acid**	Modified MITI Test conducted according to OECD TG 301 C on dl-malic acid: Readily biodegradable	Immobilization of *Daphnia magna* (OECD Guideline 202): EC_50_ >100 mg/L after 48 h exposure to fumaric acid Semi-static acute toxicity test in Juvenile fish: LC_50_ > 100 mg/L after 96 h Inhibition of total respiration in activated sludge: EC_50_ > 300 mg fumaric acid/L after 3 h	[43]
**Tartaric acid**	QSAR prediction (BioWin v4.10): Readily biodegradable	QSAR models: LC_50_ > 100 mg/L	[44]
**Phytic acid**	OECD Guideline 301A test: Degradation after 7 days was over 90% Readily biodegradable	Immobilization of *Daphnia magna* (OECD Guideline 202): EC_50_ > 294.6 µg/L after 48 h	[45]
**Mandelic acid**	OECD Guideline 301F test: Degradation after 28 days was 99% Readily biodegradable	Immobilization of *Daphnia magna* (OECD Guideline 202): EC_50_ > 100 mg/L after 48 h Algae growth inhibition (OECD Guideline 201): EC_50_ > 100 mg/L after 72 h	[46]
**Pyruvic acid**	QSAR prediction (BioWin v4.10): Readily biodegradable	No available information	[47]
**Maltobionic acid**	QSAR prediction (BioWin v4.10): Readily biodegradable	No available information	[48]
**Gluconolactone**	QSAR prediction (BioWin v4.10): Readily biodegradable	No available information	[49]

EC_50_—Effective Concentration required to produce 50% of the maximal response; LC_50_—Lethal Concentration required to kill 50% of the test organisms; QSAR—Quantitative structure activity relationship; OECD—Organization for Economic Co-operation and Development.

**Table 2:** Overview of biodegradability and acute aquatic toxicity data for vitamin C and derivatives identified in the analyzed cosmetic products.

Vitamin C Derivative	Biodegradation in Water	Aquatic Toxicity	Reference
**Ascorbic acid**	OECD Guideline 301A (Ready Biodegradability: DOC Die Away Test): Readily biodegradable after 28 days	Mortality of *Oncorhynchus mykiss* (OECD Guideline 203): LC_50_ = 1020 mg/L after 96 h Immobilization of *Daphnia magna* (OECD Guideline 202): EC_50_ = 74 mg/L after 48 h Algae growth inhibition (OECD Guideline 201): EC_50_ > 74 mg/L after 72 h	[59]
**Ascorbyl glucoside**	EU Method C.4-B (Modified OECD Screening Test): Readily biodegradable	Acute Toxicity for Daphnia (EU Method C.2): EC_50_ > 200 mg/L after 48 h Algal Inhibition test (EU Method C.3): EC_50_ > 100 mg/L after 72 h	[60]
**Ascorbyl tetraisopalmitate/tetrahexyldecyl ascorbate**	EU Method C.4-C (Carbon Dioxide Evolution Test) and OECD Guideline 301B (CO_2_ Evolution Test): Under test conditions no biodegradation observed	Immobilization of *Daphnia magna* (OECD Guideline 202): EC_50_ < 0.09 mg/L after 48 h Algae growth inhibition (OECD Guideline 201): EC_50_ > 0.09 mg/L after 72 h	[56]
**3-*O*-Ethyl ascorbic acid**	EU Method C.4-C (Carbon Dioxide Evolution Test) and OECD Guideline 301 B (CO_2_ Evolution Test): Under test conditions no biodegradation observed	Immobilization of *Daphnia magna* (OECD Guideline 202): EC_50_ > 78 mg/L after 48 h Algae growth inhibition (OECD Guideline 201): EC_50_ > 81 mg/L after 72 h	[57]
**Ascorbyl methylsilanol pectinate**	No available information	No available information	-
**Sodium ascorbyl phosphate**	92/69/EWG, C.4-D (Manom. Respirat.): Not readily biodegradable	Mortality of *Danio rerio* (zebra fish): LC_50_ = 5855.8 mg/L after 96 h Immobilization of *Daphnia magna* (OECD Guideline 202): EC_50_ > 100 mg/L after 48 h Algae growth inhibition (OECD Guideline 201): EC_50_ > 100 mg/L after 72 h *Pseudomonas putida* growth inhibition test: IC_50_ = 7700 mg/L after 16 h	[58]

EC_50_—Effective Concentration required to produce 50% of the maximal response; LC_50_—Lethal Concentration required to kill 50% of the test organisms; OECD—Organization for Economic Co-operation and Development.

**Table 3:** Overview of biodegradability and acute aquatic toxicity data for resorcinol derivatives identified in the analyzed cosmetic products.

Resorcinol Derivative	Biodegradation in Water	Aquatic Toxicity	Reference
**Butylresorcinol**	QSAR prediction: Ultimate biodegradation: Weeks Primary biodegradation: Days Anaerobic: Does not biodegrade fast Not readily biodegradable	Immobilization of *Daphnia magna* (OECD Guideline 202): EC_50_ = 0.86 mg/L after 48 h Algae growth inhibition (OECD Guideline 201): EC_50_ = 30 mg/L after 72 h	[71]
**Hexyl resorcinol**	OECD Guideline 301 D (Closed Bottle Test): Degradation after 7, 14, 21, and 28 days was 64.68, 80.85, 85.11, and 80.85% Not readily biodegradable	Immobilization of *Daphnia magna* (OECD Guideline 202): EC_50_ = 2.8 mg/L after 48 h Algae growth inhibition (OECD Guideline 201): EC_50_ = 6.24 mg/L after 72 h	[73]
**Phenylethyl resorcinol**	OECD Guideline 301 D (Closed Bottle Test): Degradation after 28 days was 1% Not readily biodegradable	Mortality of *Danio rerio* (zebra fish): LC_50_ ≥ 10, 9.65, 9.65 and 8.94 mg/L after 24, 48, 72 and 96 h Immobilization of *Daphnia magna* (OECD Guideline 202): EC_50_ = 1.41 mg/L after 48 h Algae growth inhibition (OECD Guideline 201): EC_50_ = 4.15 and 2.42 mg/L after 72 h Inhibition of total respiration in activated sludge: EC_50_ = 33 mg/L after 3 h	[72]
**Isobutylamido thiazolyl resorcinol**	OECD Guideline 301 B (CO_2_ Evolution Test): Degradation after 29 days was 33.6% Not readily biodegradable	Immobilization of *Daphnia magna* (OECD Guideline 202) EC_50_ = 16 mg/L after 48 h Algae growth inhibition (OECD Guideline 201): EC_50_ = 3.3 and 1 mg/L after 72 h	[74]

EC_50_—Effective Concentration required to produce 50% of the maximal response; LC_50_—Lethal Concentration required to kill 50% of the test organisms; QSAR—Quantitative structure activity relationship; OECD—Organization for Economic Co-operation and Development.

**Table 4:** Overview of biodegradability and acute aquatic toxicity data for each peptide and amino acid identified in the analyzed cosmetic products.

Peptide or Amino Acid	Biodegradation in Water	Aquatic Toxicity	Reference
**Undecylenoyl phenylalanine**	OECD Guideline 301 B (CO_2_ Evolution Test) and EU Method C.4-C (Carbon Dioxide Evolution Test): Degradation after 28 days was 75% Readily biodegradable	Immobilization of *Daphnia magna* (EU Method C.2 and OECD Guideline 202): EC_50_ > 110 mg/L after 48 h Algae growth inhibition (EU Method C.3 and OECD Guideline 201): EC_50_ = 89 mg/L after 72 h	[87]
**Oligopeptide-68**	QSAR prediction (BioWin v4.10): Not readily biodegradable	No available information	-
**Acetyl glycyl beta-alanine**	OECD Guideline 301 B (CO_2_ Evolution Test): Degradation after 28 days was 93.7% Readily biodegradable	Immobilization of *Daphnia magna* (EU Method C.2 and OECD Guideline 202): EC_50_ > 100 mg/L after 48 h Algae growth inhibition (EU Method C.3 and OECD Guideline 201): EC_50_ > 100 mg/L after 72 h	[88]
**Nicotiana benthamiana hexapeptide 40 SH-polypeptide 2**	No available information	No available information	-
**Hexapeptide-2**	No available information	No available information	
**Tetrapeptide-30**	QSAR prediction (BioWin v4.10): Not readily biodegradable	No available information	
**Hexanoyl dipeptide-3 norleucine acetate**	No available information	No available information	

EC_50_—Effective Concentration required to produce 50% of the maximal response; QSAR—Quantitative structure activity relationship; OECD—Organization for Economic Co-operation and Development.

**Table 5:** Overview of biodegradability and acute aquatic toxicity data for azelaic acid and its derivatives identified in the analyzed cosmetic products.

Azelaic Acid Derivative	Biodegradation in Water	Aquatic Toxicity	Reference
**Azelaic acid**	QSAR prediction and read across approach: Readily biodegradable	Algae growth inhibition (OECD Guideline 201): EC_50_ > 67 mg/L after 72 h Mortality of *Oryzias latipes* (read across approach): LC_50_ > 16 mg/L after 96 h Immobilization of *Daphnia magna* (read across approach): EC_50_ > 21 mg/L after 48 h	[96,97]
**Potassium azeloyl diglycinate**	OECD Guideline 301 B (CO_2_ Evolution Test) and EU Method C.4-C (Carbon Dioxide Evolution Test): Degradation after 28 days was between 53.7 and 65.9% Readily biodegradable	Immobilization of *Daphnia magna* (QSAR prediction): EC_50_ = 141.25 mg/L after 48 h Algae growth inhibition (QSAR prediction): EC_50_ = 102.32 mg/L after 72 h	[98]

EC_50_—Effective Concentration required to produce 50% of the maximal response; LC_50_—Lethal Concentration required to kill 50% of the test organisms; QSAR—Quantitative structure activity relationship; OECD—Organization for Economic Co-operation and Development.

**Table 6:** Overview of biodegradability and acute aquatic toxicity data for each retinoid identified in the analyzed cosmetic products.

Retinoid	Biodegradation in Water	Aquatic Toxicity	Reference
**Retinol**	OECD Guideline 301 B (CO_2_ Evolution Test): Degradation after 28 days was 81% Readily biodegradable	Mortality of *Leuciscus idus* (German national standard DIN 38 412, part L15): LC_50_ > 10,000 mg/L after 96 h Mortality of *Danio rerio* (OECD Guideline 203): LC_50_ = 316.23 mg/L after 96 h Immobilization of *Daphnia magna* (OECD Guideline 202): EC_50_ > 100 mg/L after 48 h Algae growth inhibition (German standard test guideline, DIN 38 412 part 9): EC_50_ = 152.94 mg/L after 72 h	[116]
**Retinal**	QSAR prediction: Not readily biodegradable	No available information	[115]

EC_50_—Effective Concentration required to produce 50% of the maximal response; LC_50_—Lethal Concentration required to kill 50% of the test organisms; QSAR—Quantitative structure activity relationship; OECD—Organization for Economic Co-operation and Development.

## Data Availability

Dataset available on request from the authors.

## References

[1] Zhao W., Yang A., Wang J., Huang D., Deng Y., Zhang X., Qu Q., Ma W., Xiong R., Zhu M. (2022). Potential application of natural bioactive compounds as skin-whitening agents: A review. J. Cosmet. Dermatol..

[2] Resende D.I.S.P., Ferreira M.S., Lobo J.M.S., Sousa E., Almeida I.F. (2022). Skin depigmenting agents in anti-aging cosmetics: A medicinal perspective on emerging ingredients. Appl. Sci..

[3] Smit N., Vicanova J., Pavel S. (2009). The hunt for natural skin whitening agents. Int. J. Mol. Sci..

[4] Parvez S., Kang M., Chung H.S., Cho C., Hong M.C., Shin M.K., Bae H. (2006). Survey and mechanism of skin depigmenting and lightening agents. Phytother. Res..

[5] Rendon M.I., Gaviria J.I. (2005). Review of skin-lightening agents. Dermatol. Surg..

[6] Rotava P.A., Favero J.S., Garcia K.R., Angeli V.W. (2020). Profile of depigmenting cosmetics and dermatological products on the market. Rev. Cienc. Farm. Basica Apl..

[7] Pillaiyar T., Manickam M., Namasivayam V. (2017). Skin whitening agents: Medicinal chemistry perspective of tyrosinase inhibitors. J. Enzym. Inhib. Med. Chem..

[8] Taylor A., Pawaskar M., Taylor S.L., Balkrishnan R., Feldman S.R. (2008). Prevalence of pigmentary disorders and their impact on quality of life: A prospective cohort study. J. Cosmet. Dermatol..

[9] Dlova N.C., Akintilo L.O., Taylor S.C. (2019). Prevalence of pigmentary disorders: A cross-sectional study in public hospitals in Durban, South Africa. Int. J. Womens Dermatol..

[10] Yener G., Secer A., Ghazalian P.L. (2023). What factors influence consumers to buy green products? An analysis through the motivation–opportunity–ability framework and consumer awareness. Sustainability.

[11] Cosmetics Europe – The Personal Care Association (2018). Environmental Sustainability—The European Cosmetics Industry’s Contribution 2017–2018.

[12] Milosheska D., Roškar R. (2022). Use of retinoids in topical antiaging treatments: A focused review of clinical evidence for conventional and nanoformulations. Adv. Ther..

[13] Jafry M., Guan L.L., Mohammad T.F. (2024). A practical guide to over-the-counter treatments for hyperpigmentation. JEADV Clin. Pract..

[14] Couteau C., Coiffard L. (2016). Overview of skin whitening agents: Drugs and cosmetic products. Cosmetics.

[15] Burger P., Landreau A., Azoulay S., Michel T., Fernandez X. (2016). Skin whitening cosmetics: Feedback and challenges in the development of natural skin lighteners. Cosmetics.

[16] Caritá A.C., Fonseca-Santos B., Shultz J.D., Michniak-Kohn B., Chorilli M., Leonardi G.R. (2020). Vitamin C: One compound, several uses. Advances for delivery, efficiency and stability. Nanomed. Nanotechnol. Biol. Med..

[17] Jacques C., Genies C., Bacqueville D., Tourette A., Borotra N., Chaves F., Sanches F., Gaudry A.L., Bessou-Touya S., Duplan H. (2021). Ascorbic acid 2-glucoside: An ascorbic acid pro-drug with longer-term antioxidant efficacy in skin. Int. J. Cosmet. Sci..

[18] Pintea A., Manea A., Pintea C., Vlad R.-A., Bîrsan M., Antonoaea P., Rédai E.M., Ciurba A. (2025). Peptides: Emerging candidates for the prevention and treatment of skin senescence: A review. Biomolecules.

[19] Searle T., Ali F.R., Al-Niaimi F. (2022). The versatility of azelaic acid in dermatology. J. Dermatol. Treat..

[20] Zeichner J. (2013). New insights into azelaic acid. Pract. Dermatol..

[21] Kornhauser A., Coelho S.G., Hearing V.J. (2010). Applications of hydroxy acids: Classification, mechanisms, and photoactivity. Clin. Cosmet. Investig. Dermatol..

[22] Cosmetic Ingredient Review (2013). Safety Assessment of Alpha Hydroxy Acids as Used in Cosmetics.

[23] Green B.A., Yu R.J., Van Scott E.J. (2009). Clinical and cosmeceutical uses of hydroxyacids. Clin. Dermatol..

[24] Van Scott E.J., Yu R.J. (1974). Control of keratinization with alpha-hydroxy acids and related compounds. I. Topical treatment of ichthyotic disorders. Arch. Dermatol..

[25] Ditre C.M., Griffin T.D., Murphy G.F., Sueki H., Telegan B., Johnson W.C., Yu R.J., Van Scott E.J. (1996). Effects of alpha-hydroxy acids on photoaged skin: A pilot clinical, histologic, and ultrastructural study. J. Am. Acad. Dermatol..

[26] Karwal K., Mukovozov I. (2023). Topical AHA in dermatology: Formulations, mechanisms of action, efficacy, and future perspectives. Cosmetics.

[27] Almeman A.A. (2024). Evaluating the efficacy and safety of alpha-hydroxy acids in dermatological practice: A comprehensive clinical and legal review. Clin. Cosmet. Investig. Dermatol..

[28] Freelance Formulations Exploring Common and Uncommon Acids in Skin Care. https://www.freelanceformulations.com/post/exploring-common-and-uncommon-acids-in-skin-care.

[29] Arif T. (2015). Salicylic acid as a peeling agent: A comprehensive review. Clin. Cosmet. Investig. Dermatol..

[30] Davies M., Marks R. (1976). Studies on the effect of salicylic acid on normal skin. Br. J. Dermatol..

[31] Fiume M.M., Heldreth B.A., Bergfeld W.F., Belsito D.V., Hill R.A., Klaassen C.D., Liebler D.C., Marks J.G., Shank R.C., Slaga T.J. (2014). Safety assessment of citric acid, inorganic citrate salts, and alkyl citrate esters as used in cosmetics. Int. J. Toxicol..

[32] Williams R.J.E. (2005). The Use and Safety of Hydroxy Acids in Cosmetics.

[33] Bernstein E.F., Brown D.B., Schwartz M.D., Kaidbey K., Ksenzenko S.M. (2004). The polyhydroxy acid gluconolactone protects against ultraviolet radiation in an in vitro model of cutaneous photoaging. Dermatol. Surg..

[34] Wulaningsih T.I. (2023). Gluconolactone in cosmetic. KESANS Int. J. Health Sci..

[35] Cotellesa C., Manunta T., Ghersetich I., Brazzini B., Peris K. (2004). The use of pyruvic acid in the treatment of acne. J. Eur. Acad. Dermatol. Venereol..

[36] Jankowska B., Zujko M.E. (2023). The effectiveness of pyruvic acid peeling in improving the quality of life of patients with acne vulgaris. J. Clin. Med..

[37] Tosson Z., Attwa E., Al-Mokadem S. (2006). Pyruvic acid as a new therapeutic peeling agent in acne, melasma and warts. Egypt. Dermatol. Online J..

[38] European Commission (2008). Regulation (EC) No 1272/2008 of the European Parliament and of the Council of 16 December 2008 on Classification, Labeling and Packaging of Substances and Mixtures.

[39] European Chemicals Agency (ECHA) Registration Dossier—Citric Acid. https://chem.echa.europa.eu/100.000.973/dossier-view/891540c8-350a-4291-95ce-9a577d99f6e7/7d3c8b9b-e7a7-4fd6-a232-227918bc8085_7d3c8b9b-e7a7-4fd6-a232-227918bc8085?searchText=citric%20acid.

[40] European Chemicals Agency (ECHA) Registration Dossier—Salicylic Acid. https://chem.echa.europa.eu/100.000.648/dossier-view/58350bce-5d00-4254-90f2-fc9bbae79437/04470c3b-cf4a-4b6c-adae-76df0f50e040_04470c3b-cf4a-4b6c-adae-76df0f50e040?searchText=salicylic%20acid.

[41] European Chemicals Agency (ECHA) Registration Dossier—Glycolic Acid. https://chem.echa.europa.eu/100.001.073/dossier-list/reach/dossiers/active?searchText=glycolic%20acid.

[42] European Chemicals Agency (ECHA) Registration Dossier—Lactic Acid. https://chem.echa.europa.eu/100.000.017/dossier-list/reach/dossiers/active?searchText=lactic%20acid.

[43] European Chemicals Agency (ECHA) Registration Dossier—Malic Acid. https://chem.echa.europa.eu/100.027.293/dossier-view/98f2d387-6152-4a27-89e5-7486a5627d63/9517df64-0c6b-4645-b7a2-e5d278aae26f_9517df64-0c6b-4645-b7a2-e5d278aae26f?searchText=malic%20acid.

[44] European Chemicals Agency (ECHA) Registration Dossier—Tartaric Acid. https://chem.echa.europa.eu/100.001.606/dossier-view/0cdd8531-8acb-469b-a934-c2de6fc6d515/aa654079-0621-4c29-bc7d-f2411d473f10_aa654079-0621-4c29-bc7d-f2411d473f10?searchText=tartaric%20acid.

[45] European Chemicals Agency (ECHA) Registration Dossier—Fytic Acid. https://chem.echa.europa.eu/100.001.369/dossier-list/reach/dossiers/active?searchText=phytic%20acid.

[46] European Chemicals Agency (ECHA) Registration Dossier—Mandelic Acid. https://chem.echa.europa.eu/100.001.825/dossier-view/71d849af-b8c7-4f40-95d0-60bb50b876f3/7e2da5cf-69fd-4bff-ba63-3e4ddbf3fa9f_7e2da5cf-69fd-4bff-ba63-3e4ddbf3fa9f?searchText=mandelic%20acid.

[47] ChemSpider Pyruvic Acid. https://www.chemspider.com/Chemical-Structure.1031.html?rid=58614bec-5164-465c-a3d9-64f8b4f1d6cc.

[48] ChemSpider Maltobionic Acid. https://www.chemspider.com/Chemical-Structure.2300679.html?rid=6d503220-193e-4d4c-8642-f67b23b1c479.

[49] ChemSpider D-Glucono-δ-Lactone. https://www.chemspider.com/Chemical-Structure.6760.html?rid=9f5f6f11-85e8-450c-9b13-4f5f4589c72a.

[50] Pinnell S.R., Madey D.L. (1998). Topical vitamin C in skin care. Aesthetic Surg. J..

[51] Takenouchi K., Aso K. (1964). The relation between melanin formation and ascorbic acid. J. Vitaminol..

[52] Pasonen-Seppänen S., Suhonen T.M., Kirjavainen M., Suihko E., Urtti A., Miettinen M., Hyttinen M., Tammi M., Tammi R. (2001). Vitamin C enhances differentiation of a continuous keratinocyte cell line (REK) into epidermis with normal stratum corneum ultrastructure and functional permeability barrier. Histochem. Cell Biol..

[53] Shultz J.D., Caritá A.C., Mohd H., Michniak-Kohn B., Aiello L.M., Leonardi G.R. (2022). Cosmetics formulations containing vitamin C and the instability challenge. Res. J. Top. Cosmet. Sci..

[54] Enrique Lorente P., Abdulsamed K., Volkan G. (2023). Cosmetic topical use of vitamin C. Ascorbic Acid.

[55] The Organization for Economic Cooperation and Development (OECD) (1994). SIDS (Screening Information Dataset) Initial Assessment Report.

[56] European Chemicals Agency (ECHA) Registration Dossier—(1S)-1-{(2R)-3,4-bis[(2-Hexyldecanoyl)oxy]-5-oxo-2,5-Dihydrofuran-2-yl}ethane-1,2-diyl bis(2-Hexyldecanoate). https://chem.echa.europa.eu/100.102.845/dossier-view/43ee0024-1039-4381-84e9-a62b9d5601f9/IUC5-fcb8148c-8136-4c78-ad25-bb7ac2e9f4b9_d67bee54-61f8-447d-91a9-4b97a569f8d4?searchText=183476-82-6.

[57] European Chemicals Agency (ECHA) Registration Dossier—(5R)-5-[(1S)-1,2-Dihydroxyethyl]-4-Ethoxy-3-Hydroxy-5H-Furan-2-One. https://chem.echa.europa.eu/100.123.448/dossier-view/2eb8df08-c959-4ab3-93a1-463e84584a1d/IUC5-abee2aee-d09c-49d8-8d47-79dd258c6a2f_eeecef67-49b9-4ba9-9d19-df485a8f92b6?searchText=86404-04-8.

[58] European Chemicals Agency (ECHA) Registration Dossier—Trisodium (2R)-2-[(1S)-1,2-Dihydroxyethyl]-5-oxo-4-(Phosphonooxy)-2,5-Dihydrofuran-3-Olate. https://chem.echa.europa.eu/100.102.364/dossier-view/8b7ba0f5-30f1-4002-b819-4151ce81a4be/9a7bc668-26c5-4c64-868c-bd4919670b87_d37217a3-84c3-4541-b9ca-c4474ec47344?searchText=66170-10-3.

[59] European Chemicals Agency (ECHA) Registration Dossier—Sodium Ascorbate. https://chem.echa.europa.eu/100.004.661/dossier-view/102dde58-4e3f-4444-8445-a20a5b0ace9c/16c937f8-24e2-4ad6-b450-8ab19ce8656f_9b3d6fa0-1f83-47d1-93d4-1df408f91f90?searchText=ascorbic%20acid.

[60] European Chemicals Agency (ECHA) Registration Dossier—(5R)-5-[(1S)-1,2-Dihydroxyethyl]-4-Hydroxy-3-{[(2R,3R,4S,5S,6R)-3,4,5-Trihydroxy-6-(Hydroxymethyl)oxan-2-yl]oxy}-2,5-Dihydrofuran-2-One. https://chem.echa.europa.eu/100.102.444/dossier-view/1b87bd74-2e9a-4555-902a-857bad881290/IUC5-1e1ee32a-9827-46fc-bc39-f7f5f43683d0_624d83f5-9c49-48ea-b19b-7d1c15ea86f5?searchText=Ascorbyl%20glucoside.

[61] Hakozaki T., Minwalla L., Zhuang J., Chhoa M., Matsubara A., Miyamoto K., Greatens A., Hillebrand G.G., Bissett D.L., Boissy R.E. (2002). The effect of niacinamide on reducing cutaneous pigmentation and suppression of melanosome transfer. Br. J. Dermatol..

[62] Berson D.S., Osborne R., Oblong J.E., Hakozaki T., Johnson M.B., Bissett D.L. (2013). Niacinamide. Cosmeceuticals and Cosmetic Practice.

[63] Bissett D.L., Miyamoto K., Sun P., Li J., Berge C.A. (2004). Topical niacinamide reduces yellowing, wrinkling, red blotchiness, and hyperpigmented spots in aging facial skin. Int. J. Cosmet. Sci..

[64] Monfrecola G., Gaudiello F., Cirillo T., Fabbrocini G., Balato A., Lembo S. (2013). Nicotinamide downregulates gene expression of interleukin-6, interleukin-10, monocyte chemoattractant protein-1, and tumour necrosis factor-α gene expression in HaCaT keratinocytes after ultraviolet B irradiation. Clin. Exp. Dermatol..

[65] Cosmetic Ingredient Review Expert Panel (2005). Final report of the safety assessment of niacinamide and niacin. Int. J. Toxicol..

[66] European Chemicals Agency (ECHA) Registration Dossier—Nicotinamide. https://chem.echa.europa.eu/100.002.467/dossier-view/81331048-f0b1-402b-9a98-a23603b265f8/IUC5-f534cd87-319d-4b2f-bf34-601b8e5d79a1_57b0c2ea-b7ea-4420-bdb7-ee5faf089773?searchText=98-92-0.

[67] Searle T., Al-Niaimi F., Ali F.R. (2020). The top 10 cosmeceuticals for facial hyperpigmentation. Dermatol. Ther..

[68] William W.A., Messai A.M., Penny P.G., Marcos S.-H., Mariana P.-T., Maria del Rosario G.-M. (2017). Phenolic Compounds in Water: Sources, Reactivity, Toxicity and Treatment Methods. Phenolic Compounds.

[69] Yang L.-H., Cheng H.-Y., Zhu T.-T., Wang H.-C., Haider M.R., Wang A.-J. (2021). Resorcinol as a highly efficient aromatic electron donor in bioelectrochemical system. J. Hazard. Mater..

[70] Hahn S.K., Kielhorn J., Koppenhofer J., Wibbertmann A., Mangelsdorf I. (2006). International Programme on Chemical Safety.

[71] European Chemicals Agency (ECHA) Registration Dossier—1,3-Benzenediol, 4-butyl-. https://echa.europa.eu/pt/registration-dossier/-/registered-dossier/1235/6/2/6/?documentUUID=32c84603-5929-4f70-94a4-93663d50edc6.

[72] European Chemicals Agency (ECHA) Registration Dossier—1,3-Benzenediol, 4-(1-Phenylethyl)-. https://echa.europa.eu/pt/registration-dossier/-/registered-dossier/11024.

[73] European Chemicals Agency (ECHA) Registration Dossier—4-Hexylresorcinol. https://echa.europa.eu/pt/registration-dossier/-/registered-dossier/19033.

[74] European Chemicals Agency (ECHA) Registration Dossier—Propanamide, N-[4-(2,4-Dihydroxyphenyl)-2-Thiazolyl]-2-methyl-. https://echa.europa.eu/pt/registration-dossier/-/registered-dossier/16989.

[75] Ngoc L.T.N., Moon J.-Y., Lee Y.-C. (2023). Insights into bioactive peptides in cosmetics. Cosmetics.

[76] Noguchi A., Djerassi D., Tabor A., Blair R.M. (2009). Chapter 15—Amino acids and peptides: Building blocks for skin proteins. Nutritional Cosmetics.

[77] Lintner K. (2007). Peptides, amino acids and proteins in skin care. Cosmet. Toilet..

[78] Aguilar-Toalá J.E., Hernández-Mendoza A., González-Córdova A.F., Vallejo-Cordoba B., Liceaga A.M. (2019). Potential role of natural bioactive peptides for development of cosmeceutical skin products. Peptides.

[79] Farwick M., Grether-Beck S., Marini A., Maczkiewitz U., Lange J., Köhler T., Lersch P., Falla T., Felsner I., Brenden H. (2011). Bioactive tetrapeptide GEKG boosts extracellular matrix formation: In vitro and in vivo molecular and clinical proof. Exp. Dermatol..

[80] Falla T.J., Zhang L. (2010). Efficacy of hexapeptide-7 on menopausal skin. J. Drugs Dermatol..

[81] Bauza E., Oberto G., Berghi A., Dal C.F., Domloge N. (2004). Collagen-like peptide exhibits a remarkable antiwrinkle effect on the skin when topically applied: In vivo study. Int. J. Tissue React..

[82] Fitzpatrick R.E., Rostan E.F. (2003). Reversal of photodamage with topical growth factors: A pilot study. J. Cosmet. Laser Ther..

[83] Karkouch I., Tabbene O., Gharbi D., Ben Mlouka M.A., Elkahoui S., Rihouey C., Coquet L., Cosette P., Jouenne T., Limam F. (2017). Antioxidant, antityrosinase and antibiofilm activities of synthesized peptides derived from Vicia faba protein hydrolysate: A powerful agents in cosmetic application. Ind. Crops Prod..

[84] Kubglomsong S., Theerakulkait C., Reed R.L., Yang L., Maier C.S., Stevens J.F. (2018). Isolation and identification of tyrosinase-inhibitory and copper-chelating peptides from hydrolyzed rice-bran-derived albumin. J. Agric. Food Chem..

[85] Nakchum L., Kim S.M. (2016). Preparation of squid skin collagen hydrolysate as an antihyaluronidase, antityrosinase, and antioxidant agent. Prep. Biochem. Biotechnol..

[86] Widlund H.R., Fisher D.E. (2003). Microphthalamia-associated transcription factor: A critical regulator of pigment cell development and survival. Oncogene.

[87] European Chemicals Agency (ECHA) Registration Dossier—2-(undec-10-Enoylamino)-3-Phenylpropanoic Acid. https://chem.echa.europa.eu/100.104.081/dossier-view/cadc1de7-0943-4045-a313-7bf931abad35/IUC5-bc7bcc55-a589-4829-aecc-447ff9ecbfbb_593ba7bf-7097-4842-b33d-bf1db3f6d9c2?searchText=175357-18-3.

[88] European Chemicals Agency (ECHA) Registration Dossier—3-[(2-Acetamidoacetyl)amino]Propanoic Acid. https://chem.echa.europa.eu/100.250.335/dossier-view/c8664d17-6d67-4209-b5dd-5885efb9894f/188bc598-81e2-4d82-9d09-57c04ffa2c00_89f2c4ad-d68d-4c4b-9619-c22ab912c445?searchText=1016788-34-3.

[89] King S., Campbell J., Rowe R., Daly M.-L., Moncrieff G., Maybury C. (2023). A systematic review to evaluate the efficacy of azelaic acid in the management of acne, rosacea, melasma and skin aging. J. Cosmet. Dermatol..

[90] Gupta A.K., Batra R., Bluhm R., Faergemann J. (2003). Pityriasis versicolor. Dermatol. Clin..

[91] Sauer N., Oślizło M., Brzostek M., Wolska J., Lubaszka K., Karłowicz-Bodalska K. (2023). The multiple uses of azelaic acid in dermatology: Mechanism of action, preparations, and potential therapeutic applications. Postep. Dermatol. Alergol..

[92] Schallreuter K.U., Wood J.M. (1987). Azelaic acid as a competitive inhibitor of thioredoxin reductase in human melanoma cells. Cancer Lett..

[93] Schallreuter K.U., Wood J.W. (1990). A possible mechanism of action for azelaic acid in the human epidermis. Arch. Dermatol. Res..

[94] Gollnick H. (1993). Azelaic acid-pharmacology, toxicology and mechanisms of action on keratinization in vitro and in vivo. J. Dermatol. Treat..

[95] Maramaldi G., Esposito M. (2002). Potassium azeloyl diglycinate: A multifunctional skin lightener. Cosmet. Toilet..

[96] ChemSpider Azelaic Acid. https://www.chemspider.com/Chemical-Structure.2179.html?rid=41b5624d-bc4d-4305-9cbf-aae26f0f40ba.

[97] European Chemicals Agency (ECHA) Registration Dossier—Azelaic Acid. https://chem.echa.europa.eu/100.004.246/dossier-view/565ee298-3746-4de4-904b-6aabbfe0f735/IUC5-e2cd19ff-b18a-4efb-8e6a-135efc42aabe_04eb3064-fd5c-48e1-be20-66be321bd0a8?searchText=123-99-9.

[98] European Chemicals Agency (ECHA) Registration Dossier—Glycine, N, N’—(1,9-nonandeiyl) bis-, Monopotasium Salt. https://chem.echa.europa.eu/100.104.938/dossier-view/f52d5fef-aafb-43f3-9a00-6a9c4590231c/7fc6e724-20cb-4e0e-9510-22a56b32860e_91e66f2c-c792-48af-b908-92f54803540d?searchText=Potassium%20azeloyl%20diglycinate.

[99] Taraz M., Niknam S., Ehsani A.H. (2017). Tranexamic acid in treatment of melasma: A comprehensive review of clinical studies. Dermatol. Ther..

[100] Maeda K. (2022). Mechanism of action of topical tranexamic acid in the treatment of melasma and sun-induced skin hyperpigmentation. Cosmetics.

[101] Abiko Y., Iwamoto M. (1970). Plasminogen-plasmin system: VII. Potentiation of antifibrinolytic action of a synthetic inhibitor, tranexamic acid, by α2-macroglobulin antiplasmin. Biochim. Biophys. Acta Protein Struct..

[102] Kim M.S., Bang S.H., Kim J.-H., Shin H.-J., Choi J.-H., Chang S.E. (2015). Tranexamic acid diminishes laser-induced melanogenesis. Ann. Dermatol..

[103] Horikoshi T., Eguchi H., Onodera H. (1994). The effects of tranexamic acid on the growth and melanogenesis of cultured human melanocytes. Jpn. J. Dermatol..

[104] Zhang X., Yang X., Yang H., Yang Y. (2003). Study of inhibitory effect of acidum tranexamicum on melanin synthesis. Chin. J. Dermatovenerol. Integr. Tradit. West. Med..

[105] European Chemicals Agency (ECHA) Registration Dossier—Tranexamic Acid. https://chem.echa.europa.eu/100.013.471/dossier-view/58598510-a926-4ab7-a54b-87fe23857c4d/a1f3ecb4-2961-4e92-ac77-b8d78c010f4f_ecd9d170-0b37-42a1-861f-0cbc0c117bbf?searchText=1197-18-8.

[106] Mambwe B., Mellody K.T., Kiss O., O’Connor C., Bell M., Watson R.E.B., Langton A.K. (2024). Cosmetic retinoid use in photoaged skin: A review of the compounds, their use and mechanisms of action. Int. J. Cosmet. Sci..

[107] Zasada M., Budzisz E. (2019). Retinoids: Active molecules influencing skin structure formation in cosmetic and dermatological treatments. Adv. Dermatol. Allergol..

[108] Farris P. (2022). Supplement article: Retinol: The ideal retinoid for cosmetic solutions. J. Drugs Dermatol..

[109] Reichrath J., Mittmann M., Kamradt J., Müller S.M. (1997). Expression of retinoid-X receptors (-alpha,-beta,-gamma) and retinoic acid receptors (-alpha,-beta,-gamma) in normal human skin: An immunohistological evaluation. Histochem. J..

[110] Kurlandsky S.B., Xiao J.H., Duell E.A., Voorhees J.J., Fisher G.J. (1994). Biological activity of all-trans retinol requires metabolic conversion to all-trans retinoic acid and is mediated through activation of nuclear retinoid receptors in human keratinocytes. J. Biol. Chem..

[111] Fluhr J.W., Vienne M.P., Lauze C., Dupuy P., Gehring W., Gloor M. (1999). Tolerance profile of retinol, retinaldehyde and retinoic acid under maximized and long-term clinical conditions. Dermatology.

[112] Bellemère G., Stamatas G.N., Bruère V., Bertin C., Issachar N., Oddos T. (2009). Antiaging action of retinol: From molecular to clinical. Skin Pharmacol. Physiol..

[113] Saurat J.H., Didierjean L., Masgrau E., Piletta P.A., Jaconi S., Chatellard-Gruaz D., Gumowski D., Masouyé I., Salomon D., Siegenthaler G. (1994). Topical retinaldehyde on human skin: Biologic effects and tolerance. J. Investig. Dermatol..

[114] Yeung K.W.Y., Zhou G.-J., Hilscherová K., Giesy J.P., Leung K.M.Y. (2020). Current understanding of potential ecological risks of retinoic acids and their metabolites in aquatic environments. Environ. Int..

[115] ChemSpider All-Trans-Retinal. https://www.chemspider.com/Chemical-Structure.553582.html?rid=f9afdfd3-445e-418b-9553-5977e0ddbbfd&page_num=0.

[116] European Chemicals Agency (ECHA) Registration Dossier—Retinol. https://chem.echa.europa.eu/100.000.621/dossier-view/3087b17c-dc0a-4e6b-a512-ada3ff3a689b/IUC5-82e35404-2be7-4891-9b7d-e913fe627b87_a1bbc9de-a626-4e66-a28f-c0e1d7bc9ed1?searchText=retinol.

[117] Saeedi M., Eslamifar M., Khezri K. (2019). Kojic acid applications in cosmetic and pharmaceutical preparations. Biomed. Pharmacother..

[118] Chaudhary J., Pathak A., Lakhawat S. (2014). Production technology and applications of kojic acid. Annu. Res. Rev. Biol..

[119] Phasha V., Senabe J., Ndzotoyi P., Okole B., Fouche G., Chuturgoon A. (2022). Review on the use of kojic acid—A skin-lightening ingredient. Cosmetics.

[120] Lee M., Park H.Y., Jung K.H., Kim D.H., Rho H.S., Choi K. (2020). Anti-melanogenic effects of kojic acid and hydroxycinnamic acid derivatives. Biotechnol. Bioprocess. Eng..

[121] Chen J.-K., Shen C.-R., Liu C.-L. (2010). *N*-acetylglucosamine: Production and applications. Mar. Drugs.

[122] Bissett D.L. (2006). Glucosamine: An ingredient with skin and other benefits. J. Cosmet. Dermatol..

[123] Połubinska A., Cwalinski J., Baum E., Bręborowicz A. (2013). *N*-acetylglucosamine modulates function of the skin fibroblasts. Int. J. Cosmet. Sci..

[124] Bissett D.L., Robinson L.R., Raleigh P.S., Miyamoto K., Hakozaki T., Li J., Kelm G.R. (2007). Reduction in the appearance of facial hyperpigmentation by topical N-acetyl glucosamine. J. Cosmet. Dermatol..

[125] Bissett D., Robinson L., Li J., Miyamoto K. (2006). Topical N-acetyl glucosamine reduces the appearance of hyperpigmented spots on human facial skin. J. Am. Acad. Dermatol..

[126] European Chemicals Agency (ECHA) Registration Dossier—N-acetyl-β-D-Glucosamine. https://chem.echa.europa.eu/100.028.517/dossier-view/eda3baba-6480-456f-b565-0066fe63915f/794a3344-c9d8-4bfe-8c3e-3e10c464d786_794a3344-c9d8-4bfe-8c3e-3e10c464d786?searchText=7512-17-6.

[127] Costa C., Olivi P., Botta C., Espíndola E. (2007). Toxicity in aquatic environments: Discussion and evaluation methods. Quim. Nova.

